# Supplementary material for: Identification of a binding site on soluble RANKL that can be targeted to inhibit soluble RANK-RANKL interactions and treat osteoporosis
Source: Nat Commun. 2022 Sep 12;13:5338. doi: 10.1038/s41467-022-33006-4 (PMC9468151; doi:10.1038/s41467-022-33006-4)
Supplement: Supplementary file 1 — supplementaryinformations [file 41467_2022_33006_MOESM1_ESM.pdf]

Supplementary Informations

Identification of a binding site on soluble RANKL that can be targeted to inhibit soluble RANK-RANKL interactions and treat osteoporosis

Dane Huang[1,2,3], Chao Zhao[1,3], Ruyue Li[2], Bingyi Chen[1], Yuting Zhang[1], Zhejun Sun[1], Junkang Wei[1], Huihao Zhou[1,*], Qiong Gu[1,*], Jun Xu[1,*]

[1]Research Center for Drug Discovery, School of Pharmaceutical Sciences, Sun Yat-Sen University, Guangzhou 510006, China

[2]Guangdong Provincial Key Laboratory of Research and Development in Traditional Chinese Medicine, Guangdong Provincial Second Hospital of Traditional Chinese Medicine (Guangdong Provincial Engineering Technology Research Institute of Traditional Chinese Medicine), Guangzhou 510095, China

[3]These authors contributed equally

[*]Correspondence: junxu@biochemomes.com (J.X.), guqiong@mail.sysu.edu.cn (Q.G.), zhuihao@mail.sysu.edu.cn (H.Z.)

Supplemental Table

Supplementary Table 1 Structure of S3 modified compounds and their corresponding anti-osteoclastogenesis activity IC_{50} and Cells cytotoxicity CC_{50}.

Cmpd.	Structure	IC_{50} (μM)	CC_{50} (μM)	Cmpd.	Structure	IC_{50} (μM)	CC_{50} (μM)
**S3**	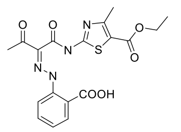	0.096	44.59	**S3-08**	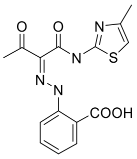	2.33	>100
**S3-00**	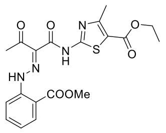	4.23	57.17	**S3-09**	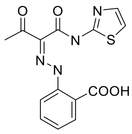	0.92	>100
**S3-01**	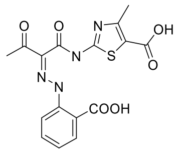	0.41	62.31	**S3-10**	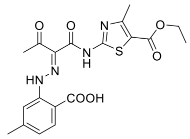	2.77	41.15
**S3-02**	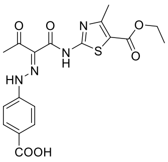	1.24	41.94	**S3-11**	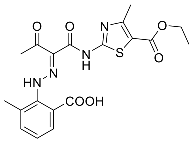	2.43	75.18
**S3-03**	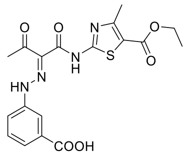	0.36	33.85	**S3-12**	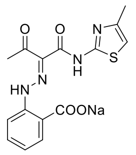	0.41	65.76
**S3-04**	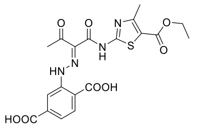	Inactive	NT	**S3-13**	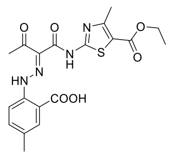	10.69	>100
**S3-05**	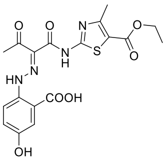	0.41	93.93	**S3-14**	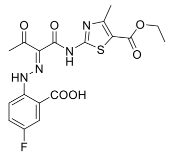	0.27	76.25
**S3-06**	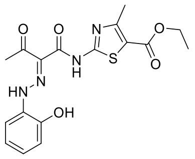	1.40	201.9	**S3-15**	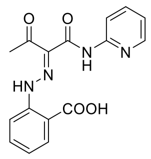	0.41	62.50
**S3-07**	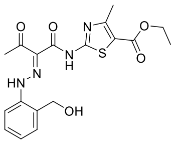	2.33	49.10	**S3-15-1**	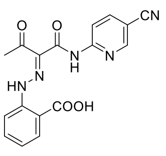	1.05	81.24

Supplementary Table 1 continued

Cmpd.	Structure	IC_{50} (μM)	CC_{50} (μM)	Cmpd.	Structure	IC_{50} (μM)	CC_{50} (μM)
**S3-15-2**	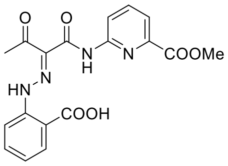	1.26	>100	**S3-18**	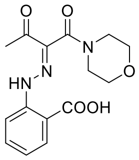	Inactive	52.58
**S3-15-3**	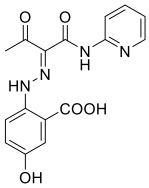	3.59	62.75	**S3-19**	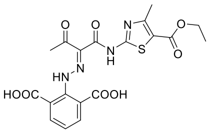	9.49	77.86
**S3-15-4**	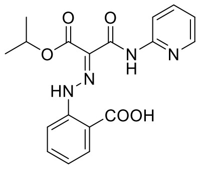	0.62	57.25	**S3-20**	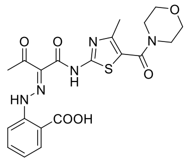	8.71	>100
**S3-16**	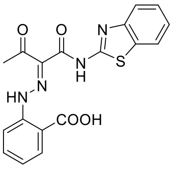	0.73	69.21	**S3-21**	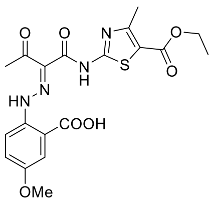	0.95	>100
**S3-17**	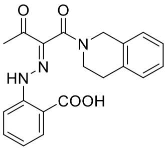	6.74	82.10	**S3-22**	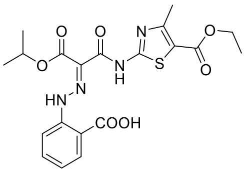	0.40	81.24

Supplementary Table 2 Structures, inhibition effects, SPR results with sRANKL and key atoms NBO charges of partial S3 series.

Cmpd.	Structure	OC inhibition (IC_{50} μM)	SPR response curves	SPR equilibrium analysis	NBO charge
**S3**	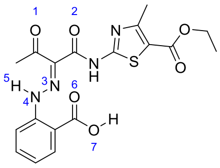	0.096	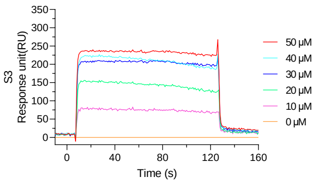	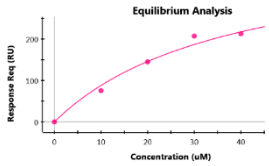<br>KD = 34.8 μM	1: -0.600<br>3: -0.121<br>6: -0.648<br>7: -0.681
**S3-01**	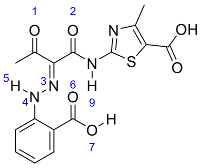	0.41	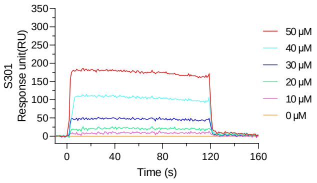	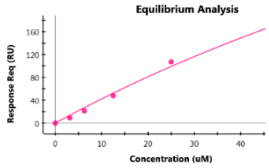<br>KD = 211 μM	1: -0.604<br>6: -0.636<br>3: -0.123<br>9: 0.440
**S3-15**	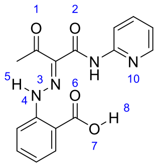	0.19	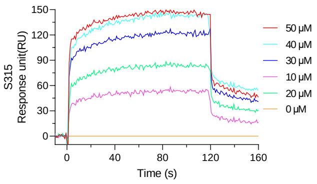	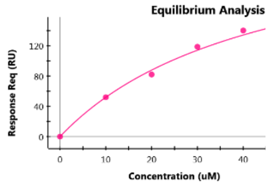<br>KD = 33.7 μM	1: -0.628<br>2: -0.674<br>7: -0.682<br>10: -0.491<br>8: 0.512
**S3-10**	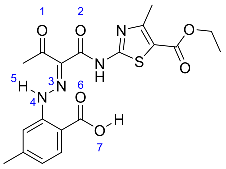	2.77	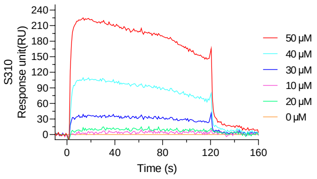	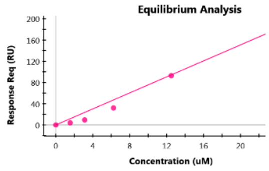<br>KD = 0.136 M	1: -0.584<br>2: -0.528
**S3-05**	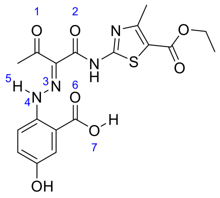	0.41	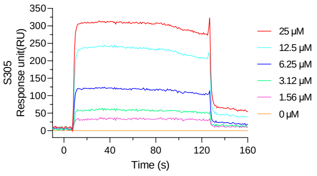	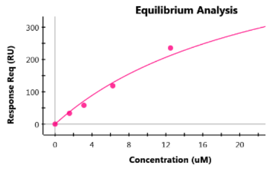<br>KD = 23.6 μM	1: -0.587<br>6: -0.631
**S3-08**	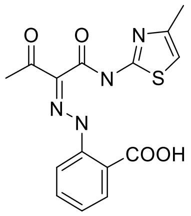	2.34	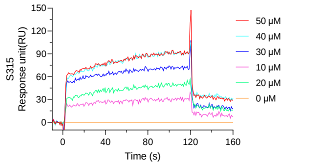	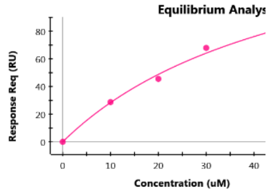<br>KD = 38.90 μM	

Supplementary Table 2 continued

Cmpd.	Structure	OC inhibition (IC_{50} μM)	SPR response curves	SPR equilibrium analysis	NBO charge
**S3-09**	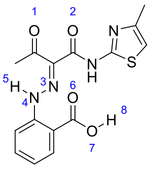	0.26	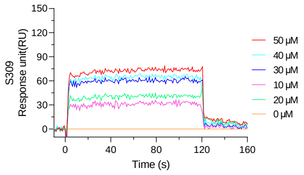	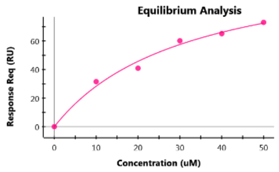 KD = 33.7 μM	1: -0.574<br>2: -0.621<br>6: -0.606<br>8: 0.5.4
**S3-11**	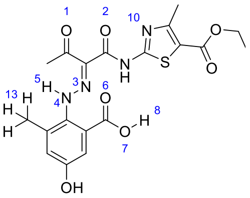	2.43	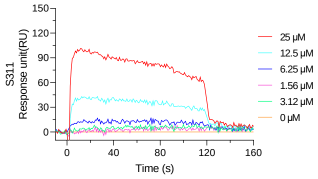	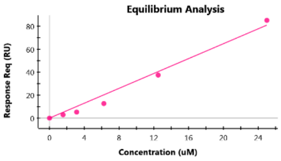 KD = 0.319 M	1: -0.582<br>10: -0.525<br>8: 0.506<br>13: 0.225
**S3-04**	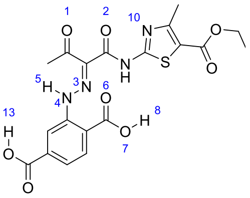	10	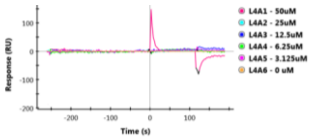	N/A	1: -0.573<br>10: -0.539<br>8: 0.509<br>13: 0.503
**S3-00**	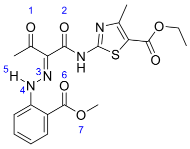	4.23	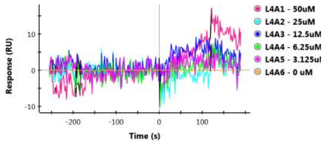	N/A	1: -0.577
**S3-07**	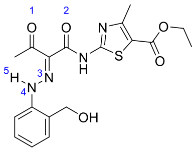	inactive	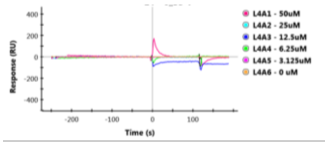	N/A	2: -0.606<br>5: 0.435
**S3-03**	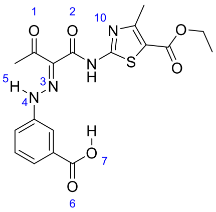	0.36	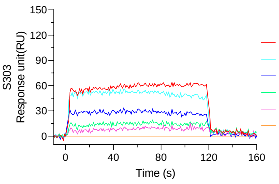	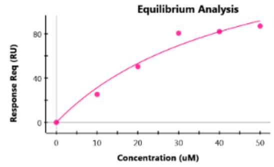 KD = 33.7 μM	1: -0.569<br>3: -0.630<br>10: -0.542
**S3-02**	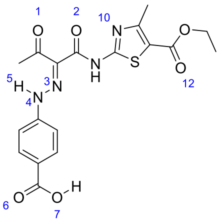	1.24	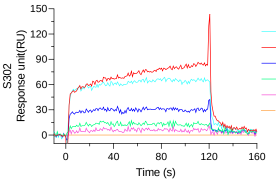	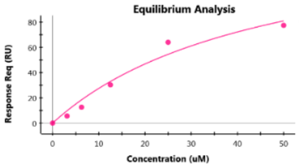 KD = 38.0 μM	1: -0.550<br>2: -0.634<br>3: -0.641<br>4: -0.689<br>12: -0.605

Supplementary Table 3. Compound **S3-15** binding proteins from cell culture supernatant.

UniProtKB	Description	Coverage [%]	# Peptides	# PSMs	# Unique Peptides	MW [kDa]	Source
O14788	Tumor necrosis factor ligand superfamily member 11（RANKL）	4	2	93	2	35.5	19 kDa band
P35979	60S ribosomal protein L12	5	1	2	1	17.8	19 kDa band
P02798	Metallothionein-2	20	1	1	1	6.1	19 kDa band
P24369	Peptidyl-prolyl cis-trans isomerase B	6	1	1	1	23.7	19 kDa band
Q61171	Peroxiredoxin-2	5	1	2	1	21.8	19 kDa band
O08807	Peroxiredoxin-4	5	1	2	1	17.8	19 kDa band

Supplementary Table4. Compound **S3-15** binding proteins from cell whole protein lysate.

UniProtKB	Description	Coverage [%]	# Peptides	# PSMs	# Unique Peptides	MW [kDa]	Source
P60867	40S ribosomal protein S20	16	2	4	2	13.4	19 kDa band
O14788	Tumor necrosis factor ligand superfamily member 11 （RANKL）	3	1	4	1	35.5	19 kDa band
Q9D358	Low molecular weight phosphotyrosine protein phosphatase	6	1	1	1	18.2	19 kDa band
Q01768	Nucleoside diphosphate kinase B	6	1	1	1	17.4	19 kDa band
Q61171	Peroxiredoxin-2	18	3	7	3	21.8	19 kDa band
P60867	40S ribosomal protein S20	16	2	4	2	13.4	19 kDa band
P16460	Argininosuccinate synthase	20	11	26	11	46.6	43 kDa band
Q8BR37	E3 ubiquitin-protein ligase NHLRC1	5	1	2	1	42.7	43 kDa band
P45952	Medium-chain specific acyl-CoA dehydrogenase, mitochondrial	3	1	2	1	46.5	43 kDa band
P09411	Phosphoglycerate kinase 1	15	4	7	4	44.5	43 kDa band
P11680	Properdin	7	2	6	2	50.3	43 kDa band
Q9JJX8	Serine/threonine-protein kinase 32B	5	1	1	1	47.9	43 kDa band
Q9Z0H3	SWI/SNF-related matrix-associated actin-dependent regulator of chromatin subfamily B member 1	3	1	2	1	44.1	43 kDa band
Q9JHJ0	Tropomodulin-3	6	2	4	2	39.5	43 kDa band
O14788	Tumor necrosis factor ligand superfamily member 11 （RANKL）	3	1	7	1	35.5	43 kDa band
A2A8Z1	Oxysterol-binding protein-related protein 9	6	4	6	4	83.1	72 kDa band
Q62148	Retinal dehydrogenase 2	2	1	1	1	56.6	72 kDa band
O35305	Tumor necrosis factor receptor superfamily member 11A	2	1	1	1	66.6	72 kDa band
O08784	Treacle protein	2	2	4	2	134.9	130 kDa band

Supplementary Table 5. Compound **S3-15** specific binding proteins from rat serum.

UniProtKB	Description	Coverage [%]	# Peptides	# PSMs	# Unique Peptides	MW [kDa]	Source
B0BNJ1	LOC683667 protein	15	3	5	3	21.6	19 kDa band
A0A140TAC5	60S ribosomal protein L12	5	1	1	1	17.9	19 kDa band
P31399	ATP synthase subunit d, mitochondrial	7	1	1	1	18.8	19 kDa band
G3V794	Tumor necrosis factor ligand superfamily member （RANKL）	13	6	56	6	35.3	19 kDa band
P27139	Carbonic anhydrase 2	7	2	4	2	29.1	26 kDa band
G3V794	Tumor necrosis factor ligand superfamily member （RANKL）	18	6	50	6	35.3	26 kDa band
P29975	Aquaporin-1	4	1	1	1	28.8	26 kDa band
P00406	Cytochrome c oxidase subunit 2	3	1	1	1	25.9	26 kDa band
G3V913	Heat shock 27kDa protein 1	10	2	2	2	22.8	26 kDa band
P57113	Maleylacetoacetate isomerase	15	3	3	3	23.9	26 kDa band
F1M8Z6	TNF receptor superfamily member 11A	2	1	1	1	67.6	26 kDa band
F1LTN6	Uncharacterized protein	42	11	144	10	24.9	26 kDa band
A0A0G2K3Z9	Uncharacterized protein	15	3	7	3	22.2	26 kDa band
A0A0G2K3K2	Actin, cytoplasmic 1	3	1	1	1	42.1	55 kDa band
G3V794	Tumor necrosis factor ligand superfamily member	15	4	17	4	35.3	55 kDa band
A0A0G2K477	Uncharacterized protein	17	6	16	3	51.1	55 kDa band

Supplemental Figures

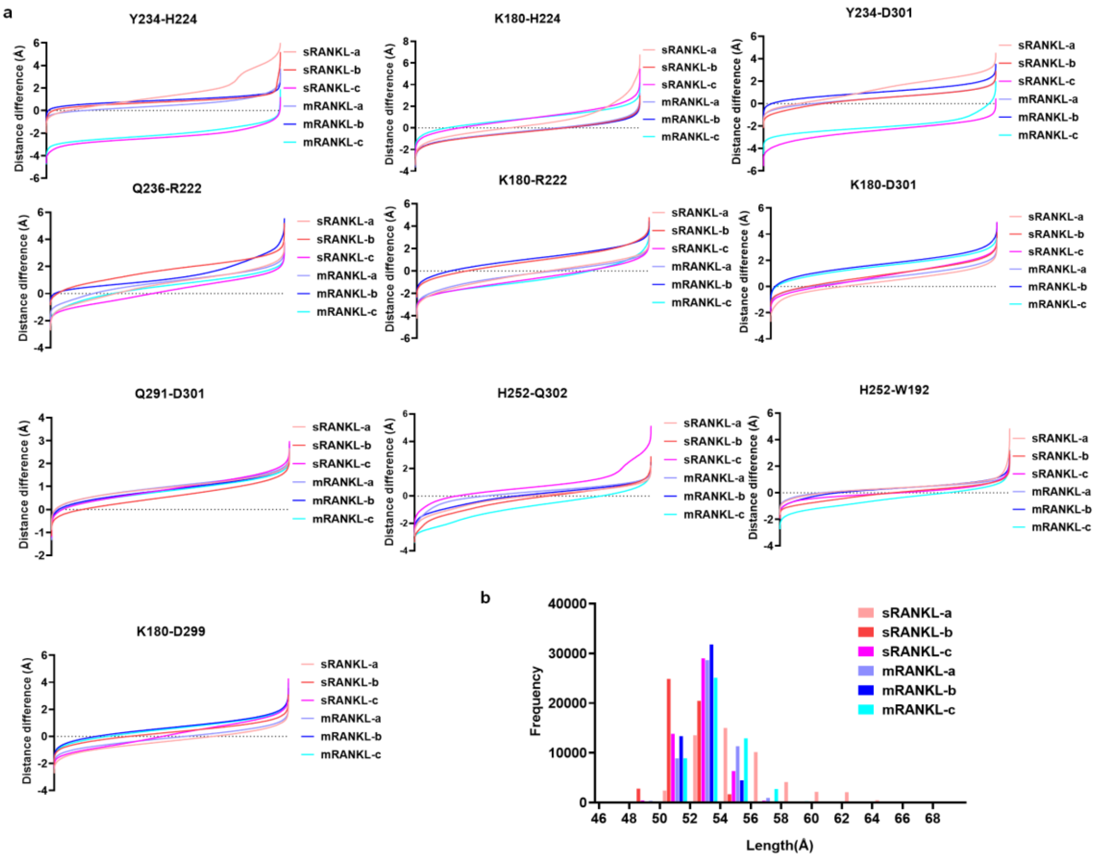

Supplementary Fig 1. Molecular Simulation for The Identify of mouse sRANKL Selective Binding Pocket.

a The distance change between residues of Y234- H224, K180-H224, Y234-D301, Q236-R222, K180-R222, K180-D301, Q291-D301, H252-Q302, H252-W192, and K180-D299 in mouse sRANKL (PDB ID: 1S55) during 100 ns MD simulation at the fix and free state. The distance change = distance in sRANKL or fixed-RANKL- the distance in crystal structure (PDB ID: 1S55). The number of distance change is aligned from small to large. Overall, most of them in both sRANKL and fixed-RANKL were getting larger. H252-Q302, H252-W192 in both sRANKL and mRANKL were maintained. In Y234- H224, Y234-D301 and Q236-R222, sRANKL exhibited larger changing than mRANKL in most of times. **b** The frequency of distance summation of Y234-H224, K180-H224, Y234-H224 and Y234-D303 of mouse sRANKL (PDB ID: 1S55) in free or fixed state during 100 ns MD simulation.

a1

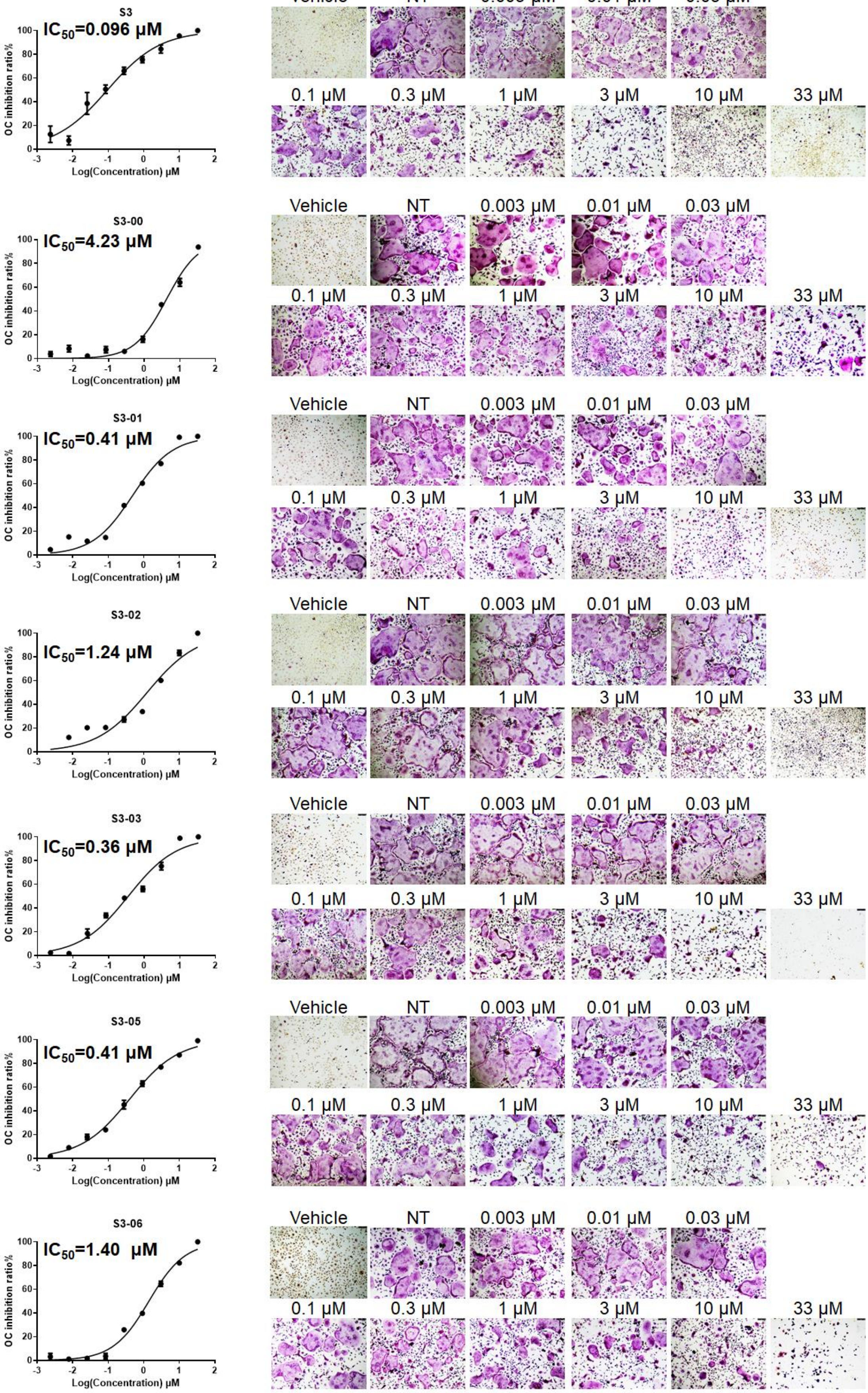
S3
IC50=0.096 μM
S3-00
IC50=4.23 μM
S3-01
IC50=0.41 μM
S3-02
IC50=1.24 μM
S3-03
IC50=0.36 μM
S3-05
IC50=0.41 μM
S3-06
IC50=1.40 μM
OC inhibition ratio%
Log(Concentration) μM
Vehicle
NT
0.003 μM
0.01 μM
0.03 μM
0.1 μM
0.3 μM
1 μM
3 μM
10 μM
33 μM

a2

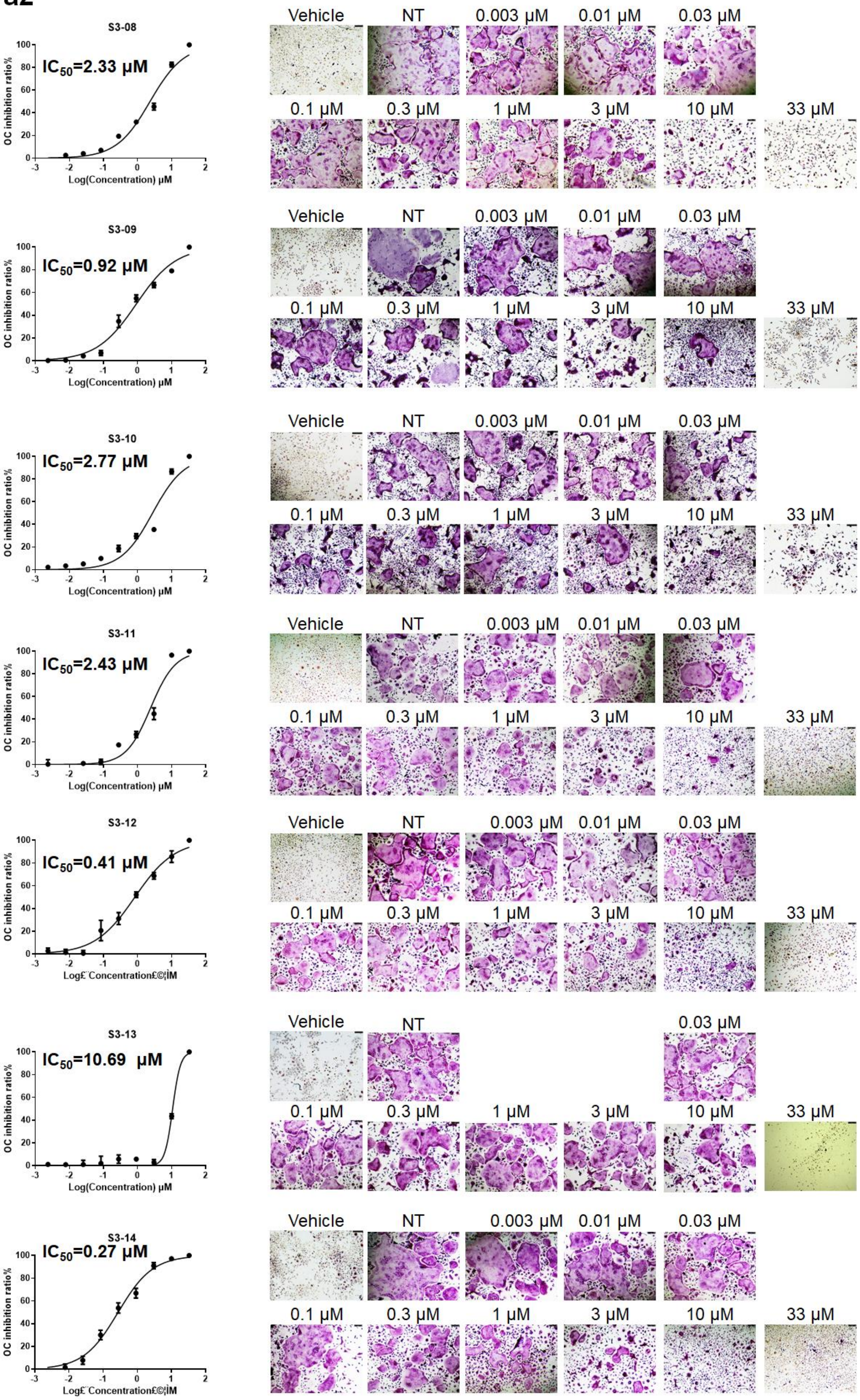
S3-08
IC50=2.33 μM
S3-09
IC50=0.92 μM
S3-10
IC50=2.77 μM
S3-11
IC50=2.43 μM
S3-12
IC50=0.41 μM
S3-13
IC50=10.69 μM
S3-14
IC50=0.27 μM
OC inhibition ratio%
Log(Concentration) μM
Log£¨Concentration£©¦ÌM
Vehicle
NT
0.003 μM
0.01 μM
0.03 μM
0.1 μM
0.3 μM
1 μM
3 μM
10 μM
33 μM

a3

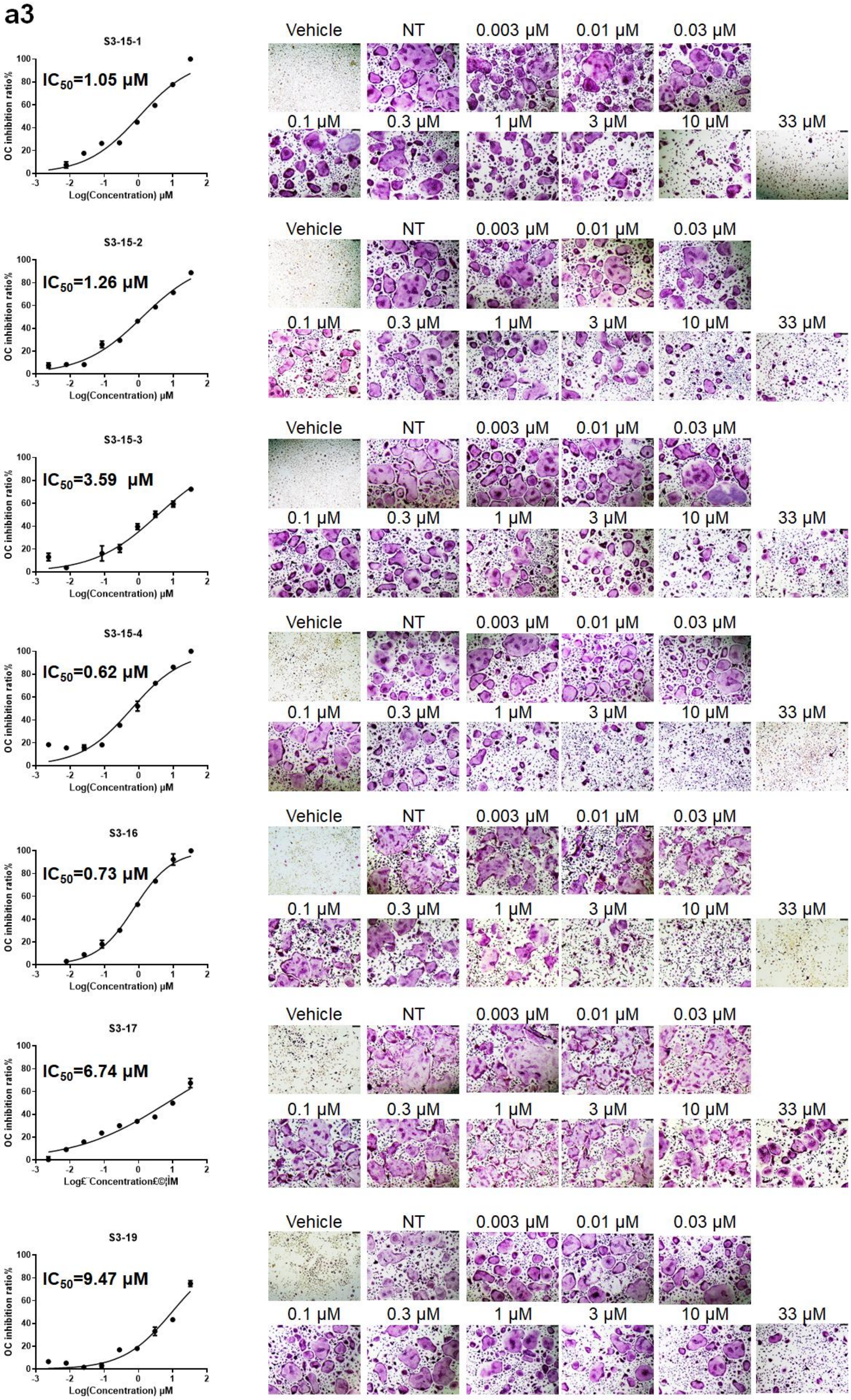
S3-15-1
IC50=1.05 μM
S3-15-2
IC50=1.26 μM
S3-15-3
IC50=3.59 μM
S3-15-4
IC50=0.62 μM
S3-16
IC50=0.73 μM
S3-17
IC50=6.74 μM
S3-19
IC50=9.47 μM
OC inhibition ratio%
Log(Concentration) μM
Vehicle
NT
0.003 μM
0.01 μM
0.03 μM
0.1 μM
0.3 μM
1 μM
3 μM
10 μM
33 μM

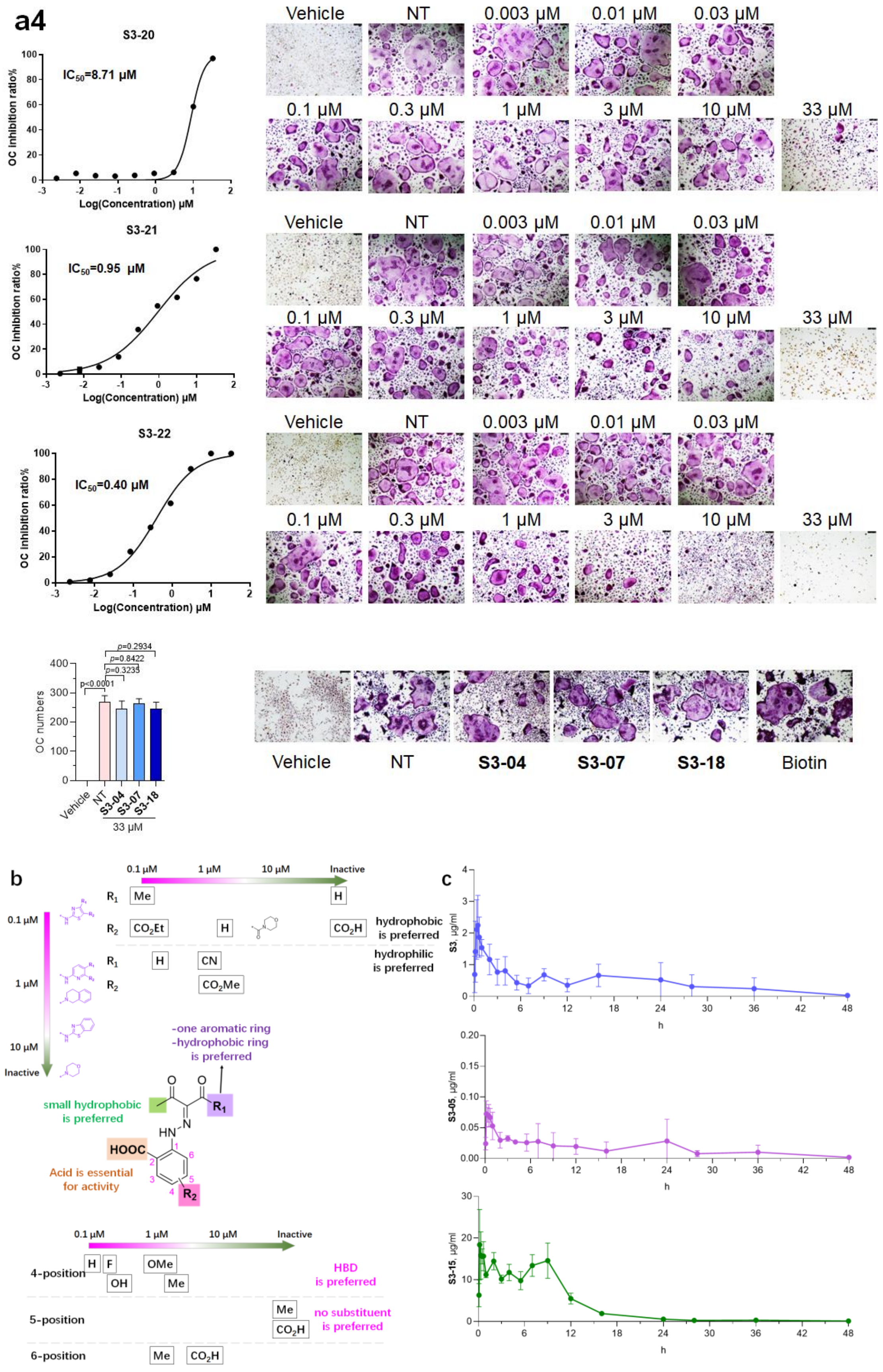

Supplementary Fig. 2. Activity, Cytotoxicity, Binding Affinity, Structure-Activity Relationship Analysis, and Pharmacokinetics Study.

a (1-4) IC_{50} curve and representative OC microphotographs of S3 modified active compounds. Left: IC_{50} curve of S3 modified compounds. Right: representative microphotographs of multinucleated TRAP-positive osteoclasts from each group. IC_{50} values were calculated by GraphPad Prism 8 software. **b** Summary of structure-activity relationship studies of S3 compound in osteoclast differentiation and apoptosis assays. A single aromatic ring is more suitable for R1 substitution. When isoquinoline (S3-17) and benzothiazole ring (S3-16) is applied, the activity is decreased compare to thiazole (S3, S3-08) or pyridine ring (S3-15). Moreover, the morpholine ring (S3-18) totally lost activity. Therefore, a single aromatic ring is more preferred. Increasing hydrophilicity of thiazole ring like removing Me group (S3-09) or introducing morpholine (S3-20) ring significantly decreased the activity, well the carboxylic acid group (S301) totally lost activity. Both hydrophilic group (S3-15-1) and hydrophobic group (S3-15-2) in pyridine ring affects the activity little. This data further indicated that the size of R1 substituent may be important for activity, hydrophobic or large substituent decrease the activity. For R2 substituent, a hydrogen bond donor such as OH (S3-05) or F (S3-14) at 4-position can maintain the activity. All the other groups decrease or lost activity. The carboxylic acid group at 2-position is essential for activity, changing the substituted position (S3-02 and S3-03) all affect the activity. Changing acid to OH (S3-06) or CH_2OH (S3-07) also suppress the inhibition effects. Larger substitution (S3-22 and S3-15-4) on the position of methyl group (green box) will decrease the activity. A small group seems to be preferred. **c** Pharmacokinetics of S3, s3-05, and S3-15. Male SD rats were dosed orally with S3, s3-05, and S3-15 at 10 mg/kg. Concentration in the plasma within 48 hr were detected by HPLC-MS. Compartmental pharmacokinetics parameters were assessed using WinNonlin Pharmacokinetics software. Data are presented as the mean ± s.d. n= n=3 for each group. Statistical difference was determined by unpaired two-tailed Student's t test. Significant difference *p* value is < 0.05.

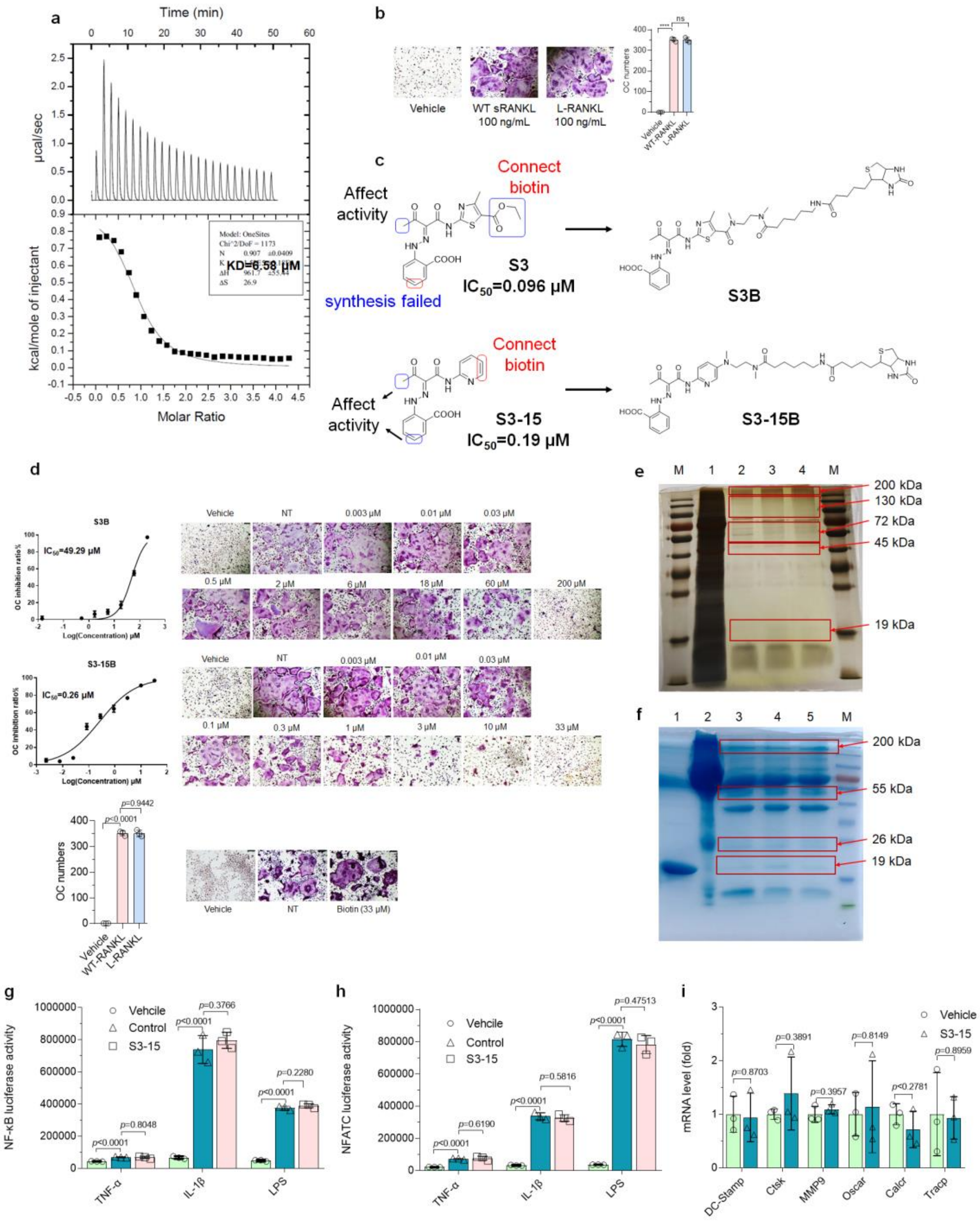

Supplementary Fig.3. The Selectivity of Compounds.

a Binding affinity of compound S3-05 to sRANKL by ITC assay with KD value of 6.58 μM. b Osteoclastogenesis activity of L-RANKL and WT sRANKL. **c** Designed of biotin probe. The red box represents that introducing group may not affect activity. The blue box represents that introducing group will decrease activity significantly. According to the SAR study, one synthesis was success and afforded compound **S3-15B**. Considering introducing large group or hydrophilic group can decrease the activity (see also supplementary Fig. 2b), the biotin is introduced into thiazole ring of S3 which gave inactive probe **S3B**. **d** Anti-osteoclastogenesis activity of biotin and its conjugate compounds, **S3B** and **S3-15B**. Left: OC inhibition IC_{50} curve and number of TRAP-positive multinucleated osteoclasts of biotin. Right: representative pictures of TRAP-positive multinucleated osteoclasts for each group. **e** Target enrichment of **S3B** and **S3-15B** by using ABPP assay. Rat serum was loaded onto the positive (**S3-15B**) linked, negative (**S3B**) linked and control (B) columns. Lane M, protein marker ladder; lane 1, sRANKL; lane 2, rat serum; line 3, biotin; line 4, **S3-**

15B; line 5, **S3B**. The bands indicated by arrowheads are differential bands that used for protein mass spectrometry analyzing. **f** Target enrichment of **S3B** and **S3-15B** by using ABPP assay. Cell lysate was loaded onto the positive (**S3-15B**) linked, negative (**S3-15B**) linked and control (B) columns. Lane M, protein marker ladder; lane 1, cell lysate; lane 2, biotin; line 3, **S3B**; line 4, **S3-15B**. The bands indicated by arrowheads are differential bands that used for protein mass spectrometry analyzing. **g-h S3-15** cannot suppress TNF-α, IL-β and LPS induced NF-ķB and NFATC activity. **i** In BMMs cells without RANKL, **S3-15** exhibited no differ with control group. Error bars represent ± s.d. n=3 for b, d, g-i. Statistical difference was determined by unpaired two-tailed Student's t test (b and d). Statistical difference was determined by one-way ANOVA followed by Tukey's multiple comparisons test. h-i. Significant difference *p* value is < 0.05. Scale bars for b and d, 50 μm.

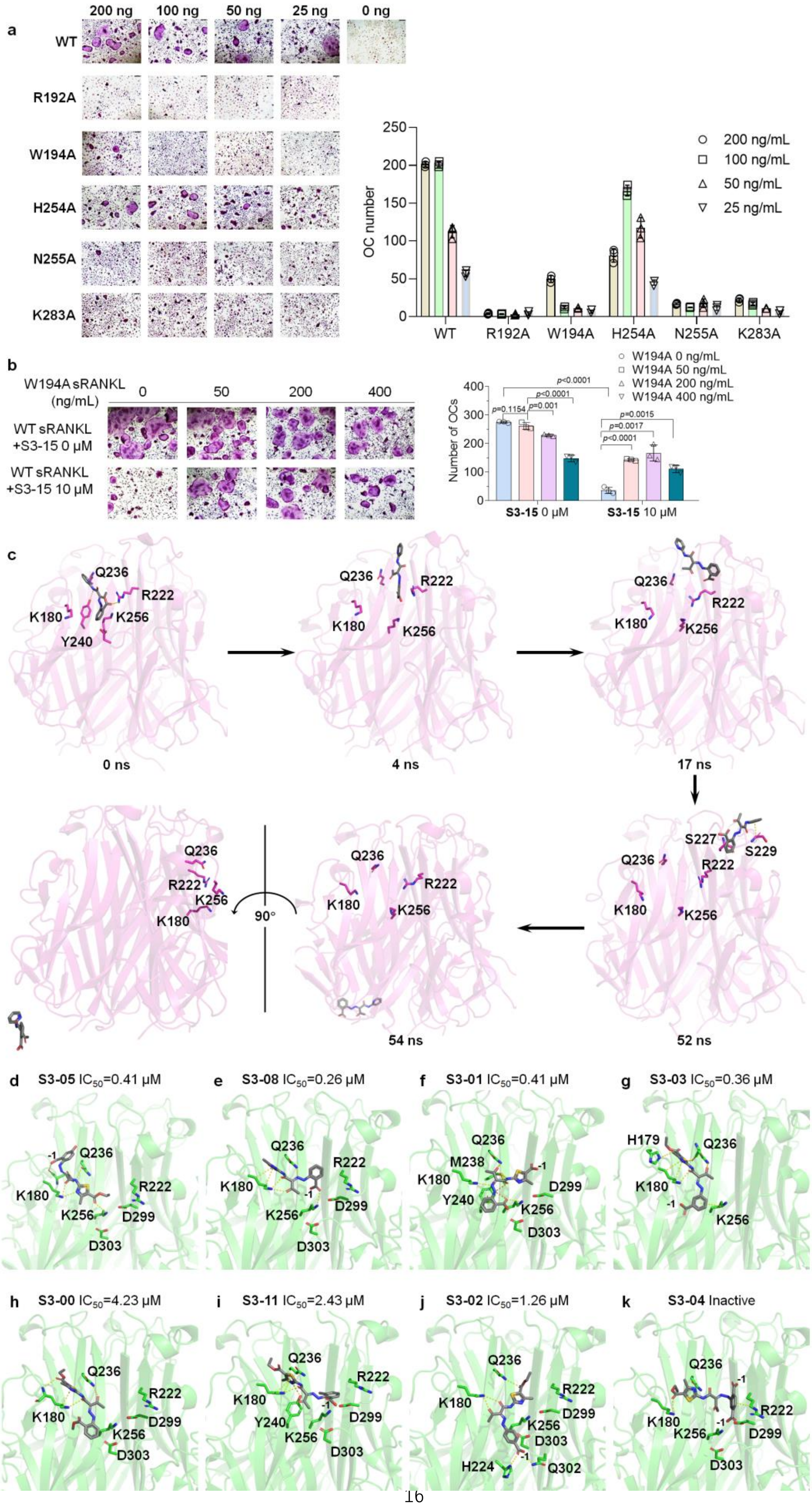

a
200 ng
100 ng
50 ng
25 ng
0 ng
WT
R192A
W194A
H254A
N255A
K283A
OC number
200 ng/mL
100 ng/mL
50 ng/mL
25 ng/mL
WT
R192A
W194A
H254A
N255A
K283A
b
W194A sRANKL (ng/mL)
0
50
200
400
WT sRANKL +S3-15 0 μM
WT sRANKL +S3-15 10 μM
Number of OCs
W194A 0 ng/mL
W194A 50 ng/mL
W194A 200 ng/mL
W194A 400 ng/mL
p=0.1154
p<0.0001
p=0.001
p<0.0001
p=0.0015
p=0.0017
p<0.0001
S3-15 0 μM
S3-15 10 μM
c
Q236
R222
K180
K256
Y240
0 ns
4 ns
17 ns
S227
S229
52 ns
54 ns
90°
d S3-05 IC50=0.41 μM
e S3-08 IC50=0.26 μM
f S3-01 IC50=0.41 μM
g S3-03 IC50=0.36 μM
K180
Q236
R222
K256
D299
D303
M238
Y240
H179
h S3-00 IC50=4.23 μM
i S3-11 IC50=2.43 μM
j S3-02 IC50=1.26 μM
k S3-04 Inactive
H224
Q302

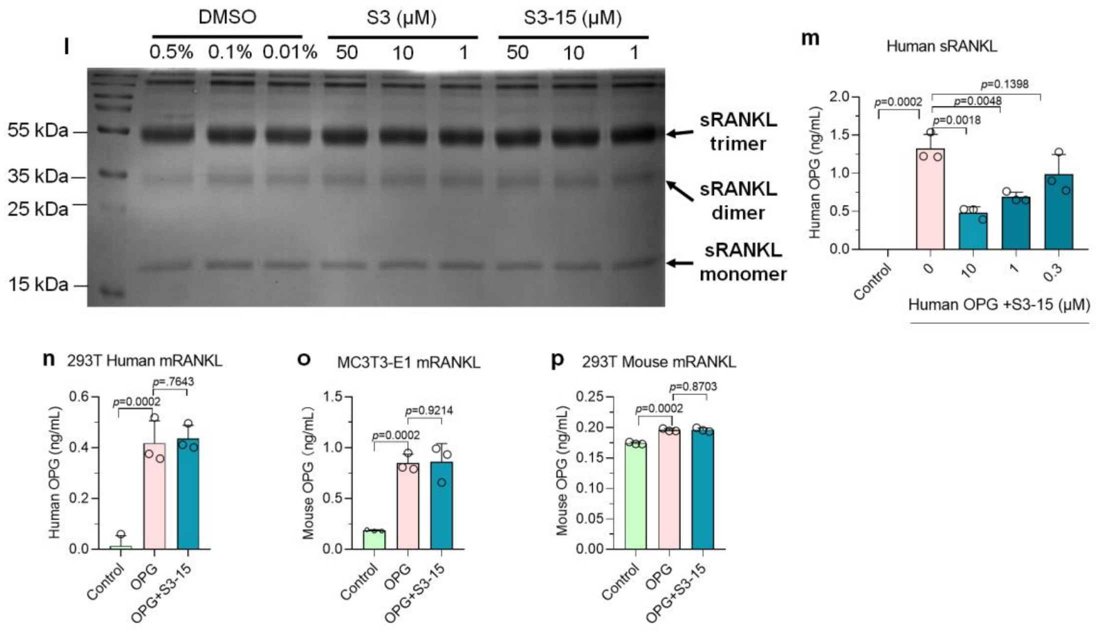

Supplementary Fig. 4. Binding Site Identified and S315 Binding Mode Analyzed.

a Representative TRAP staining images (left) of mouse BMMs cultured with different mutant sRANKL or WT sRANKL. Osteoclast numbers were counted (right). n = 3 per group. Scale bars, 50 µm. **b** In WT sRANKL-induced osteoclastogenesis system, the anti-osteoclastogenesis activity of **S3-15** is reduced when W194A sRANKL added (left, OC TRAP-stained pictures taken from one of three biological replicates. right, the OC numbers were counted in each well and presented in mean±s.d. Unpaired two-tailed Student's t test. n = 3 per group. **c** The MD simulation process of **S3-15** with fixed-RANKL. After 4 ns MD simulation, **S3-15** moved to upside and lost all H-bonds with fixed-RANKL. Then **S3-15** kept moving upside at 17ns and 52ns. At 52ns, **S3-15** already moved to the tips of fixed-RANKL, and formed two *p*-π conjugation interactions with S229 and S231. At 54ns, **S3-15** totally lost binding with fixed-RANKL and away from it. **d-k** The binding model of **S3-05**, **S3-08**, **S3-01**, **S3-03, S3-00**, **S3-11**, **S3-02**, and **S3-04. l** Cross-link assay results that identifying whether **S3** and **S3-15** affect sRANKL trimerization. Both cannot affect sRANKL trimerization in different concentrations. **m** S3-15 influenced sRANKL binds to OPG at high concentration of 10 and 1 μM. However, it cannot affects sRANKL and OPG binding at lower concentration of 0.3 μM. **n S3-15** has no effects on human mRANKL expressed on MC3T3 cell lines and human OPG binding. **o S3-15** has no effects on mouse mRANKL expressed on MC3T3 cell lines and mouse OPG binding. **p** The results of pull-down assay that evaluating whether **S3-15** affects and mouse OPG binding. **S3-15** has no effects on mouse mRANKL expressed on 293T cell lines and mouse OPG binding. Error bars represent ± s.d. n=3. Statistical difference was determined by one-way ANOVA followed by Tukey's multiple comparisons test. Significant difference *p* value is < 0.05.

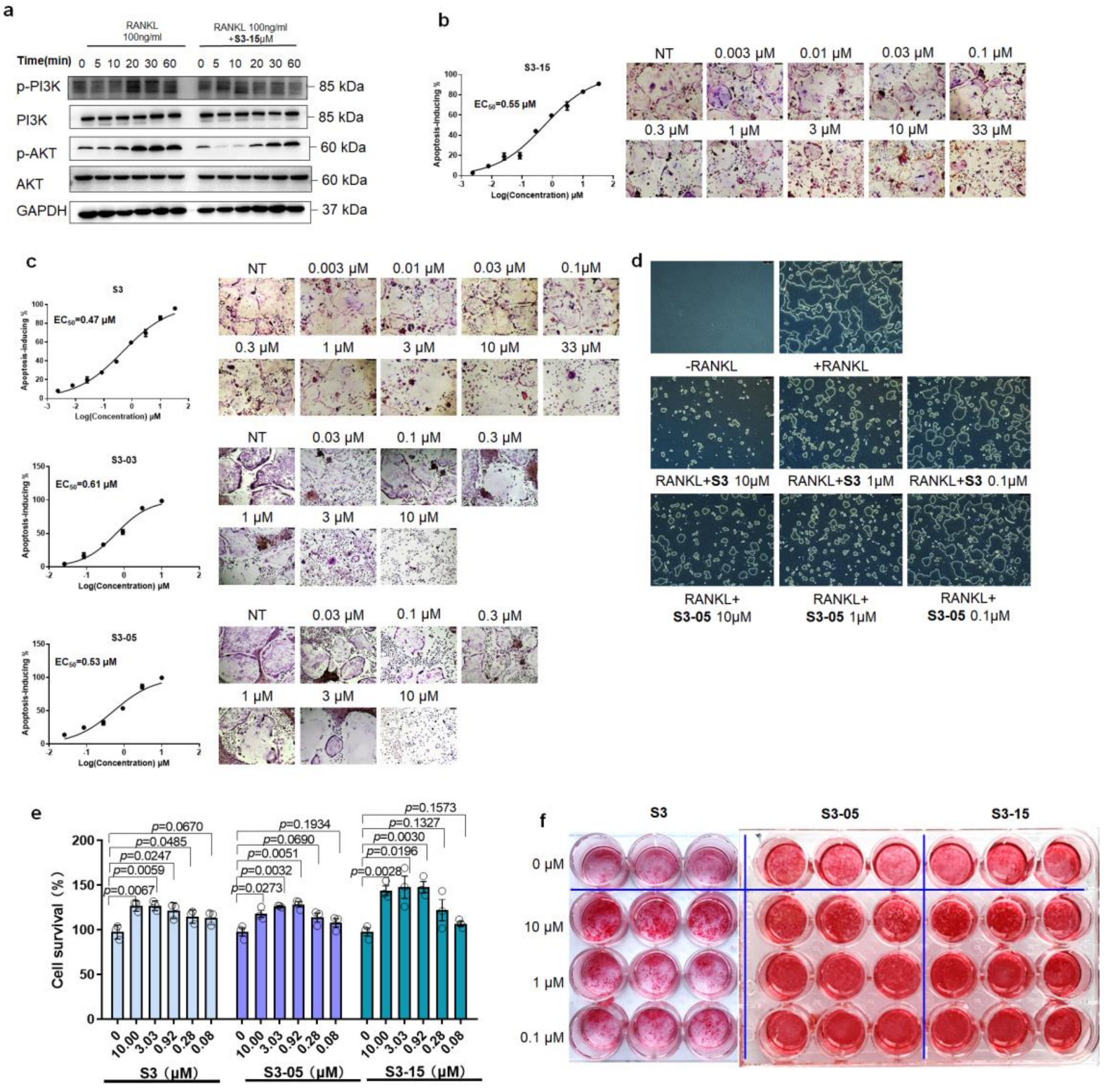

Supplementary Fig.5. In Vitro Activity Evaluation and Signal Pathway Study of compound S3-15.
a Western blot of PI3K and AKT phosphorylation in RAW264.7 cells treating with or without S315 and sRANKL at different point-in-time. **b** EC_{50} curve and representative OC microphotographs of mature osteoclasts apoptosis assay with **S3-15**. n=3 biological replicates for each concentration. EC_{50} values were calculated by GraphPad Prism 8 software. **c** EC_{50} curve and representative OC microphotographs of mature osteoclasts apoptosis assay with **S3**, **S3-03** and **S3-05**. n=3 biological replicates for each concentration. EC_{50} values were calculated by GraphPad Prism 8 software. **d** Bone resorption experimental results show that **S3** and **S3-05** suppresses RANKL-induced resorption pits in dose-dependent manner (mouse BMMs). **e** Precursor osteoblasts C3H10T12 proliferation-promoting of **S3**, **S3-05** and **S3-15**. **f** Osteoblastic mineralization after treatment with **S3**, **S3-05** and **S3-15**. C3H10T12 cells were cultured in osteoblast induction differentiation medium with or without treatment for 14 days and determinate of calcium deposition in differentiated osteoblasts by Alizarin red staining. Error bars represent ± s.d. n=3. Statistical difference was determined by unpaired two-tailed Student’s t test. Significant difference p value is < 0.05. Scale bars for b-d, 50 μm.

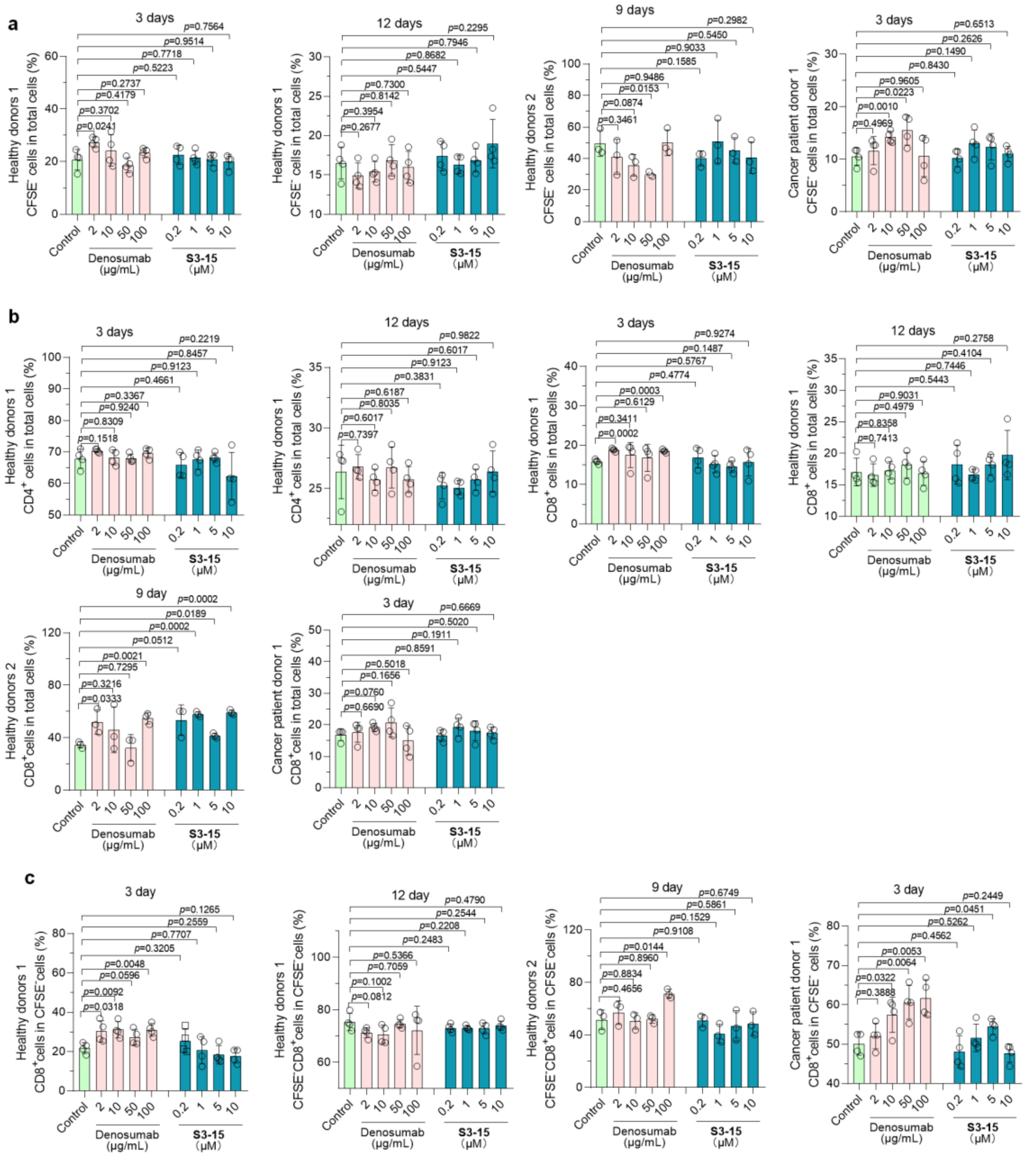

Supplementary Fig. 6. Lymphocyte proliferation and differentiation effects of S315 and denosumab.

a The effects of **S3-15** and denosumab on lymphocyte proliferation. Lymphocyte isolated from two healthy donors and one cancer patient donor were treated with **S3-15** or denosumab for 3 days, 9 days or 12 days. The ratio of CFSE-cells were detected by flow cytometry and calculated to evaluate lymphocyte proliferation. **b** The effects of **S3-15** and denosumab on T lymphocyte differentiation in total cells. After treating with **S3-15** or denosumab for 3 days, 9 days or 12 days, CD4+ T cell and CD8+ T cell were detected by flow cytometry and calculated the ratio in total cells. **c** The effects of **S3-15** and denosumab on T lymphocyte differentiation in CFSE- cells. Ratio of CD4+ T cell and CD8+ T cell in gate of CFSE- cells were analyzed after flow cytometry. Error bars represent ± s.d. n=3 for healthy donors 2. n=4 for healthy donors 1 and cancer patient donor 1. Statistical difference was determined by unpaired two-tailed Student's t test. Significant difference *p* value is < 0.05.

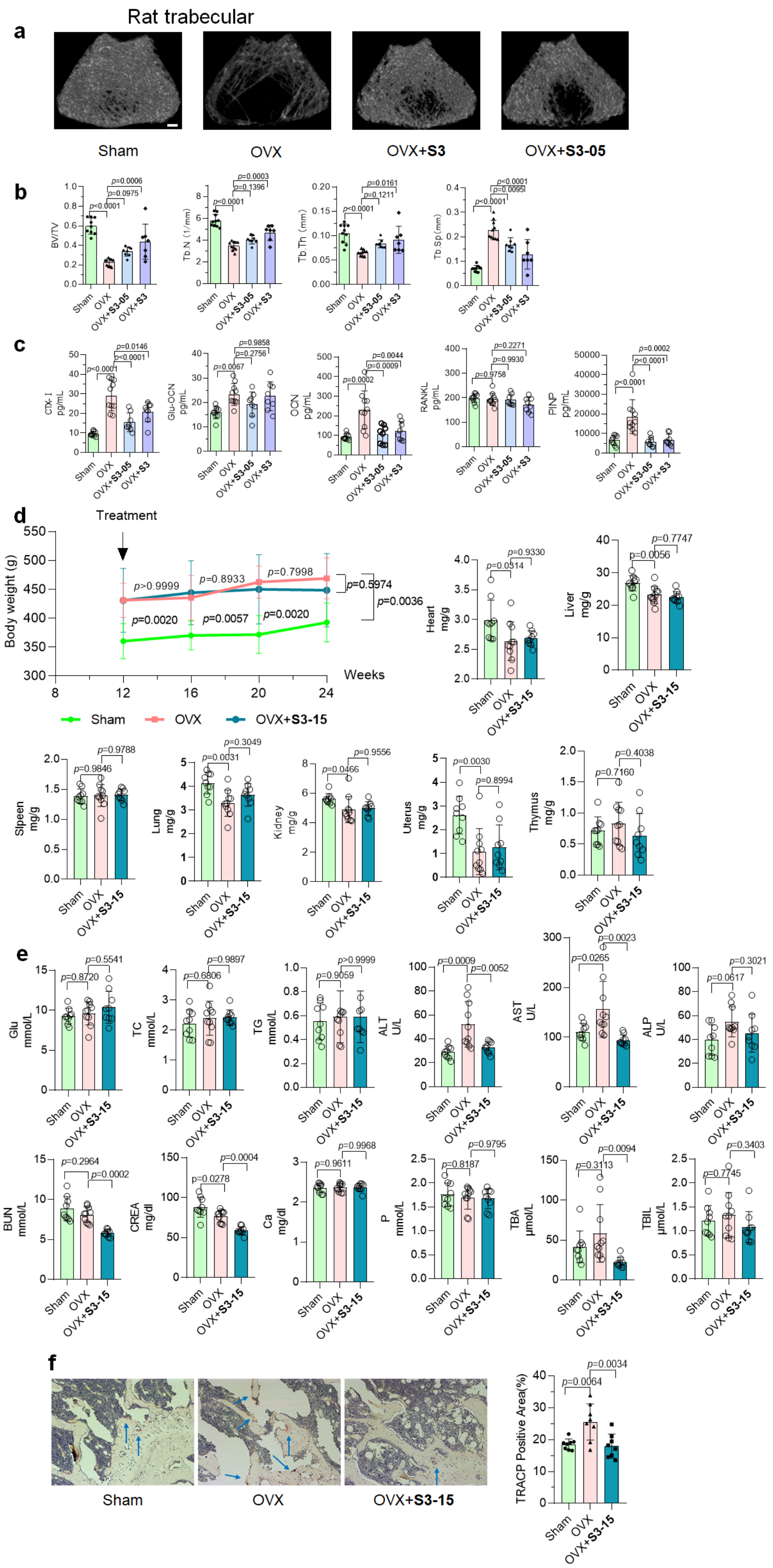

a
Rat trabecular
Sham
OVX
OVX+S3
OVX+S3-05
b
BV/TV
Tb N (1/mm)
Tb Th (mm)
Tb Sp (mm)
c
CTX-I pg/mL
Glu-OCN pg/mL
OCN pg/mL
RANKL pg/mL
PINP pg/mL
d
Treatment
Body weight (g)
Weeks
p>0.9999
p=0.8933
p=0.7998
p=0.5974
p=0.0036
p=0.0020
p=0.0057
p=0.0020
Sham
OVX
OVX+S3-15
Heart mg/g
Liver mg/g
Slpeen mg/g
Lung mg/g
Kidney mg/g
Uterus mg/g
Thymus mg/g
e
Glu mmol/L
TC mmol/L
TG mmol/L
ALT U/L
AST U/L
ALP U/L
BUN mmol/L
CREA mg/dl
Ca mg/dl
P mmol/L
TBA μmol/L
TBIL μmol/L
f
Sham
OVX
OVX+S3-15
TRACP Positive Area(%)
p=0.0064
p=0.0034

Supplementary Fig. 7. Analysis of S3, S305, and S315 Activity, Toxicity In Vivo.

a Compound **S3** and **S3-05** reduces bone loss in OVX rats. Representative μCT images of the trabecular of ROI in distal femur of rat sham, OVX or treated with **S3** and **S3-05**. Scalar bar, 500μm. **b** Compound **S3** and **S3-05** improve trabecular osteoporosis parameters in OVX rats. The value of BV/TV, Tb.Th, Tb.N in OVX rats treating with 10mg/kg/d **S3** and **S3-05** were significantly increase, while the value of Tb.Sp were significantly decrease. **c** Serum bone resorption marker CTX-I, Glu- osteocalcin, and RANKL or bone formation marker osteocalcin and PINP levels measured by ELISA. **d** In vivo toxicity evaluation of compound **S3-15**. The weight of the rats was recorded every four weeks. At the end of the treatment, heart, liver, spleen, lung, kidney, uterus, thymus was weighted and calculate their organ coefficients to examine the effects of **S3-15** on rats. **e** Serum Ca, P, AST, ALT, ALP, CREA, BUN, Glu, TC, TG, TBIL, and TBA level were also determined. **f** Histological examination of tissue sections from the femur of mice using TRAP staining. The osteoclasts were stained in red (left). Trap positive area was calculated (right). The result indicated that **S3-15** significantly inhibits osteoclast differentiation compare to OVX. The number of rats in each group is as follows: sham n=9, OVX n=10, OVX+S3-05 n=8, OVX+S3 n=7, S3-15 n=9. The number of mice is n=8 in each group. Data are expressed as the mean ± s.d. Statistical difference was determined by one-way ANOVA followed by Tukey's multiple comparisons test. Significant difference *p* value is < 0.05.

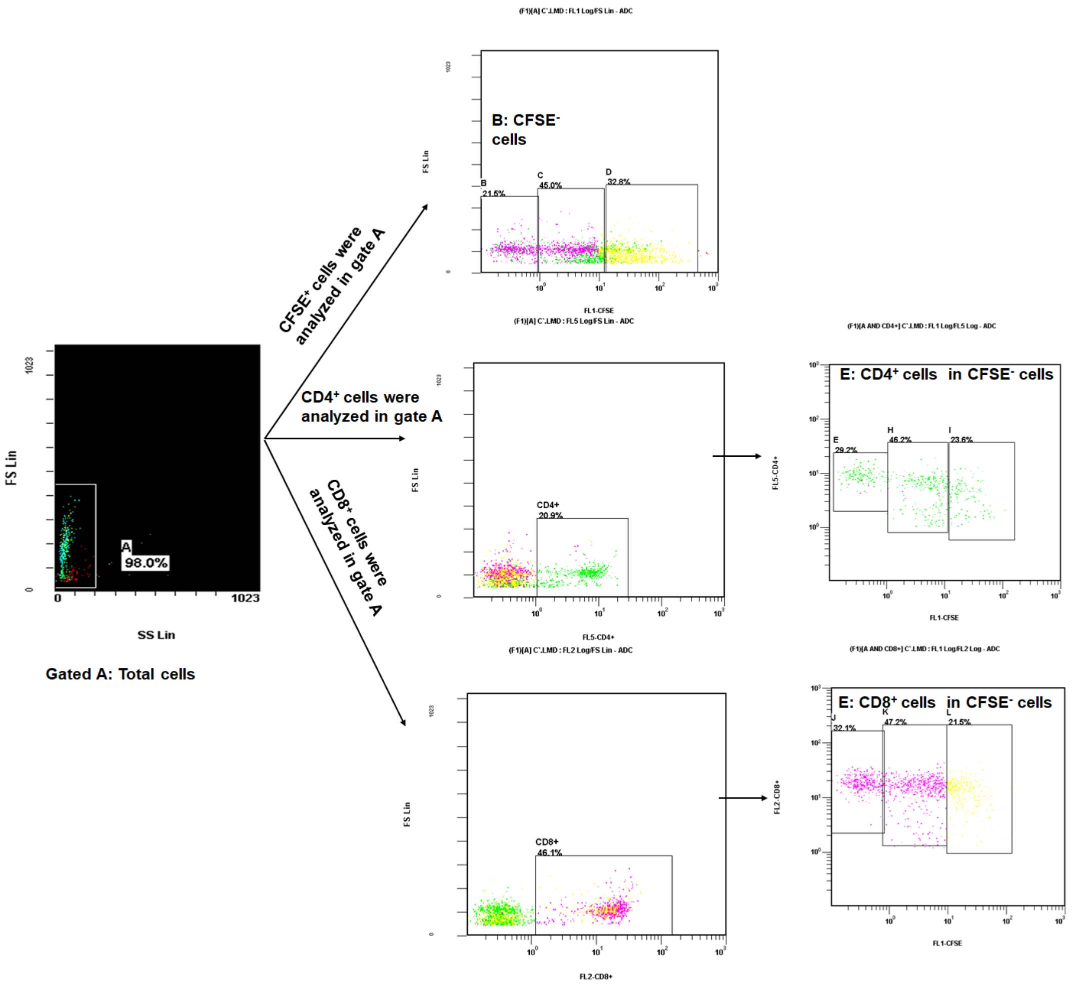

Supplementary Fig. 8. Gating Strategy for Flow Cytometric analyses of CFSE, CD4$^+$ and CD8$^+$ in human T lymphocyte cells.

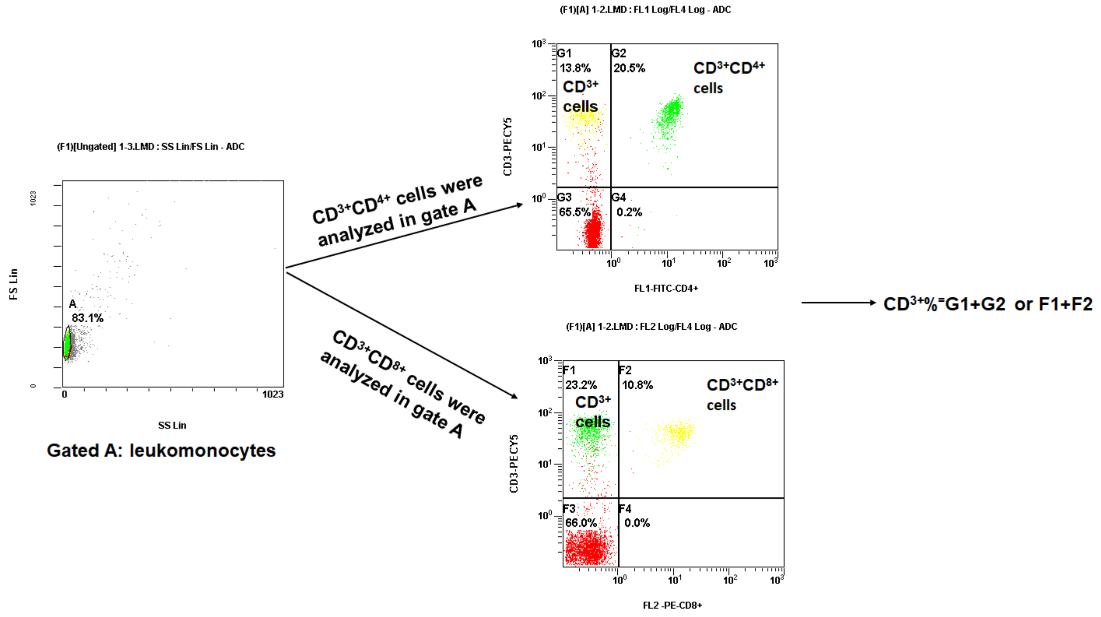

Supplementary Fig. 9. Gating Strategy for Flow Cytometric analyses of CD^{3+}, CD3$^+$CD4$^+$ and CD3+CD8$^+$ in mouse peripheral blood.

KEY RESOURCES TABLE

REAGENT or RESOURCE	SOURCE	IDENTIFIER
Antibodies		
Rabbit monoclonal anti-Phospho-NF-κB p65 (Ser536) antibody	Cell Signaling Technology	Cat# 3033; RRID: AB_331284
Rabbit monoclonal anti-NF-κB p65 NF-κB p65 antibody	Cell Signaling Technology	Cat# 8242; RRID: AB_10859369
Rabbit monoclonal anti-Phospho-p44/42 MAPK (Erk1/2) (Thr202/Tyr204) antibody	Cell Signaling Technology	Cat# 4370; RRID: AB_2315112
Rabbit monoclonal anti-p44/42 MAPK (Erk1/2) antibody	Cell Signaling Technology	Cat# 4695; RRID: AB_390779
Rabbit monoclonal anti-Phospho-IκBα (Ser32) (14D4) antibody	Cell Signaling Technology	Cat# 2859; RRID: AB_561111
Rabbit monoclonal anti- IκBα antibody	Cell Signaling Technology	Cat# 4812; RRID: AB_10694416
Rabbit monoclonal anti-Phospho-SAPK/JNK antibody (Thr183/Tyr185) (81E11) antibody	Cell Signaling Technology	Cat# 4668; RRID: AB_823588
Rabbit monoclonal anti-SAPK/JNK antibody	Cell Signaling Technology	Cat# 9252; RRID: AB_2250373
Rabbit monoclonal anti-Phospho-Akt (Ser473) antibody	Cell Signaling Technology	Cat# 9271; RRID: AB_329825
Rabbit monoclonal-anti-pan-AKT antibody	Abcam	Cat# ab8805; RRID: AB_306791
Rabbit monoclonal anti-PI 3 Kinase p85 alpha (phospho Y607) antibody	Abcam	Cat# ab182651; RRID: AB_2756407
Rabbit monoclonal anti-PI 3 Kinase p85 alpha antibody	Abcam	Cat# ab191606
Rabbit monoclonal anti-beta actin antibody	Abcam	Cat# ab8226; RRID: AB_306371
Bacterial		
Escherichia coli DH5 alpha cells	This study	N/A
Escherichia coli BL21 (DE3) cells	This study	N/A
Chemicals and Recombinant Proteins		
Fetal Bovine Serum (Mammalian cell culture)	Bovogen	Cat# 1803A
Penicillin-Streptomycin (Mammalian Cell Culture)	Gibco	Cat# 15140122
MEM-alpha basic (Medium for mammalian cell culture)	Gibco	Cat# C12641800BT
DMEM (Medium for mammalian cell culture)	Gibco	Cat# C11965500BT
Murine M-CSF	Peprotech	Cat# 315-02
In Vivo Ready anti-Human CD28 (CD28.2)	Tonbo Biosciences	Cat# 40-0289-U500
In Vivo Ready anti-Human CD3 (OKT3)	Tonbo Biosciences	Cat# 40-0037-U500
Human IL-2	Peprotech	Cat# 200-02
GST-RANKL	This study	N/A
RANKL	This study	N/A
L-RANKL	This study	N/A
RANK	This study	N/A

Biotin	Energy chemical	Cat# E080117; CAS: 58-85-5
Human osteoprotegerin, Fc tag	Acrobiosytems	Cat# TNB-H5259-100 μg
Mouse osteoprotegerin, Fc tag	SinoBiological	Cat# 52613-M02H
**S3**	SPEC	XOS00045
Critical Commercial Assays		
Acid Phosphatase, Leukocyte (TRAP) Kit	Sigma	Cat# 387A
RevertAidTM First Strand cDNA Synthesis Kit	Thermo Fisher	Cat# K1622
Maxima SYBR Green/Fluorescein qPCR Master Mix (2X)	Thermo Fisher	Cat# K0241
Annexin V-FITC Apoptosis Detection Kit	Bevotime	Cat# C1062L
T4 ligase	Merck	69839
PCR reaction Kit	TOYOBO	QPK-201
Plasmid Midiprep Kit	QIAGEN	12145
PE-Cyanine7 anti-Human CD8a (RPA-T8)	Tonbo Biosciences	Cat# 60-0088-T100
PE anti-Human CD4 (RPA-T4)	Tonbo Biosciences	Cat# 50-0049-T100
PerCP-Cyanine5.5 anti-Human CD3 (OKT3)	Tonbo Biosciences	Cat# 65-0037-T100
ELISA Kit for Human Osteoprotegerin	Wuhan USCN Business	Cat# SEA108Hu
ELISA Kit for Mouse Osteoprotegerin	Wuhan USCN Business	Cat# SEA108Mu
REAGENT or RESOURCE		
Ni-NTA agarose resin	QIAGEN	30210
Glutathione Agarose Resin	Merck	G4510
Streptavidin–Agarose from Streptomyces avidinii	Sigma	Cat# S1638
Carboxyfluorescein succinimidyl amino ester (CFSE)	Tonbo Biosciences	Cat# 13-0850-U500
Chemical characterizations	This study	N/A
Experimental Models: Cell Lines		
Mouse: RAW264.7 cells	ATCC	Cat# TIB-71, RRID: CVCL_0493
Mouse: RAW264.7 cells stably transfected with an NF-κB-driven luciferase reporter gene construct (3kB-Luc-SV40)	JiaKe Xu Lab	N/A
Mouse: RAW264.7 cells stably transfected with an NFATc1 luciferase reporter construct	JiaKe Xu Lab	N/A
Human: HEK293T	ATCC	Cat# CRL-3216, RRID: CVCL_0063
BMMs	This study	N/A
Experimental Models: Organisms/Strains		
C57BL/6 (Female)	Guangdong Medical Laboratory Animal Center	N/A
Sprague Dawley rat (SD, Female)	Guangdong Medical Laboratory Animal Center	N/A
Oligonucleotides		
DC-stamp F:5'-GGGGACTTATGTGTTTCCACG-3'	Sangon Biotech	N/A
DC-stamp R:5'-ACAAAGCAACAGACTCCCAAAT-3'	Sangon Biotech	N/A
Calcr F: 5'-TGCAGACAACTCTTGGTTGG-3'	Sangon Biotech	N/A
Calcr R:5'-TCGGTTTCTTCTCCTCTGGA-3'	Sangon Biotech	N/A

Oscar F: 5′-CTCTTCAAAAGTGGCCTTGTCA-3′	Sangon Biotech	N/A
Oscar R: 5′-GGAAGAACTCAGCCAGCTCAA-3′	Sangon Biotech	N/A
Tracp F: 5′-GGCTATGTGCTGAG-3′	Sangon Biotech	N/A
Tracp R: 5′- GGAGGCTGGTCTTA-3′	Sangon Biotech	N/A
β-actin F: TCCAGCCTTCCTTCTTGGGTAT	Sangon Biotech	N/A
β-actin R: TGTTGGCATAGAGGTCTTTACGG	Sangon Biotech	N/A
Ctsk F: CAGCAGAACGGAGGCATTGA	Sangon Biotech	N/A
Ctsk R:CCTTTGCCGTGGCGTTATAC	Sangon Biotech	N/A
MMP9 F: CCTACTCTGCCTGCACCACTAAA	Sangon Biotech	N/A
MMP9 R: CTGCTTGCCCAGGAAGACGAA	Sangon Biotech	N/A
Software and Algorithms		
MOE 2018	Chemical Computing Group ULC	N/A
Pymol 2.3.4	Schrödinger	N/A
ChemBioOffice 2014	CambridgeSoft	N/A
Schrödinger 2018-1	Schrödinger	N/A
Amber 16	AMBER Software Administrator	N/A
GraphPad Prism 8	GraphPad Software	N/A
ImageJ	NIH	N/A
ProteOn XPR36 ProteonManager	Bio-Rad	N/Ac

The sequences of sRANKL and mRANKL mimics

The amino acid sequence of sRANKL was the C-terminal extracellular region of RANKL (rat 159-318 aa). The amino acid sequence of mRANKL was two sRANKL monomers linked by -Gly-Ser-Gly-Ser-.

(1) sRANKL (rat 159-318 aa)

GKPEAQPFAHLTINAANIPSGSHKVSLSSWYHDRGWAKISNMTLSNGKLRVNQDGFYYLYANICFRHHETSGS
VPADYLQLMVYVVKTSIKIPSSHNLMKGGSTKNWSGNSEFHFYSINVGGFFKLRAGEEISVQVSNPSLLDPDQD
ATYFGAFKVQDID

(2) GST-sRNAKL (rat 159-318 aa)

GKPEAQPFAHLTINAANIPSGSHKVSLSSWYHDRGWAKISNMTLSNGKLRVNQDGFYYLYANICFRHHETSGS
VPADYLQLMVYVVKTSIKIPSSHNLMKGGSTKNWSGNSEFHFYSINVGGFFKLRAGEEISVQVSNPSLLDPDQD
ATYFGAFKVQDID

(3) mRANKL (sRANKL-GSGS-sRANKL, rat 159-318 aa)

GKPEAQPFAHLTINAANIPSGSHKVSLSSWYHDRGWAKISNMTLSNGKLRVNQDGFYYLYANICFRHHETSGS
VPADYLQLMVYVVKTSIKIPSSHNLMKGGSTKNWSGNSEFHFYSINVGGFFKLRAGEEISVQVSNPSLLDPDQD
ATYFGAFKVQDIDGSGSGKPEAQPFAHLTINAANIPSGSHKVSLSSWYHDRGWAKISNMTLSNGKLRVNQDG
FYYLYANICFRHHETSGSVPADYLQLMVYVVKTSIKIPSSHNLMKGGSTKNWSGNSEFHFYSINVGGFFKLRAG
EEISVQVSNPSLLDPDQDATYFGAFKVQDID

Compounds synthesis

(*E*)-2-(2-(1-((5-(ethoxycarbonyl)-4-methylthiazol-2-yl) amino)-1,3-dioxobutan-2-ylidene) hydrazinyl) benzoic acid: S3

S3 was synthesized according to the general procedure as yellow solid with 58% yield.

^{1}H NMR (500 MHz, DMSO-d_6) δ 12.84 (bs, 1H), 7.98 (d, *J* = 10.0 Hz, 1H), 7.80 (d, *J* = 10.0 Hz, 1H), 7.46 (t, *J* = 10.0 Hz, 1H), 7.16 (t, *J* = 10.0 Hz, 1H), 4.26 (q, *J* = 5.0 Hz, 2H), 2.56 (s, 3H), 2.54 (s, 3H), 1.32 (t, *J* = 5.0 Hz, 3H).

^{13}C NMR (126 MHz, DMSO-d_6) δ162.56, 157.01, 131.90, 131.42, 114.51, 60.97, 26.45, 17.44, 14.70.

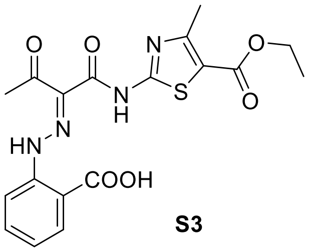

S3

Ethyl (E)-2-(2-(2-(2-(methoxycarbonyl)phenyl)hydrazono)-3-oxobutanamido)-4-methylthiazole-5-carboxylate: S3-00

S3-00 was synthesized according to the general procedure as yellow solid with 45% yield.

^{1}H NMR (400 MHz, $CDCl_3$) δ 15.35 (s, 1H), 12.50 (s, 1H), 8.04 (d, *J* = 8.0 Hz, 1H), 7.89 (d, *J* = 8.0 Hz, 1H), 7.56 (t, *J* = 8.0 Hz, 1H), 7.18 (t, *J* = 8.0 Hz, 1H), 4.26 (q, *J* = 4.0 Hz, 2H), 4.03 (s, 3H), 2.60 (s, 3H), 2.56 (s, 3H), 1.31 (t, *J* = 4.0 Hz, 3H).

^{13}C NMR (100 MHz, $CDCl_3$) δ 198.17, 165.68, 161.82, 159.37, 156.78, 156.00, 142.11, 133.45, 130.70, 125.01, 123.72, 115.69, 115.52, 114.82, 59.83, 51.83, 25.18, 16.29, 13.40.

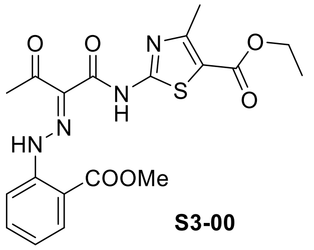

S3-00

(E)-2-(2-(2-(2-carboxyphenyl)hydrazono)-3-oxobutanamido)-4-methylthiazole-5-carboxylic acid: S3-01

S3-01 was synthesized according to the general procedure as yellow solid with 92% yield.

^{1}H NMR (500 MHz, DMSO-d_6) δ 15.06 (d, *J* = 20.0 Hz, 1H), 12.50 (d, *J* = 15.0 Hz, 1H), 8.02-7.96 (m, 2H), 7.73 (d, *J* = 10.0 Hz, 1H), 7.30 (t, *J* = 10.0 Hz, 1H), 3.80 (s, 3H), 2.50 (s, 3H).

^{13}C NMR (126 MHz, DMSO-d_6) δ 168.52, 162.73, 143.49, 135.08, 131.84, 125.04, 117.04, 115.96, 52.44, 26.42, 17.38.

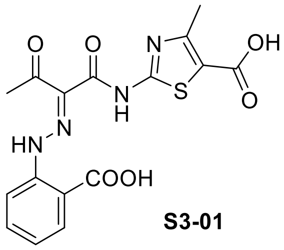

S3-01

(E)-4-(2-(1-((5-(ethoxycarbonyl)-4-methylthiazol-2-yl)amino)-1,3-dioxobutan-2-ylidene)hydrazinyl)benzoic acid: S3-02

S3-02 was synthesized according to the general procedure as yellow solid with 58% yield.

^{1}H NMR (500 MHz, DMSO-d_6) δ 12.95 (bs, 1H), 7.98 (d, *J* = 10.0 Hz, 2H), 7.61 (d, *J* = 10.0 Hz, 2H), 4.26 (q, *J* = 5.0 Hz, 2H), 2.57 (s, 3H), 2.51 (s, 3H), 1.30 (t, *J* = 5.0 Hz, 3H).

^{13}C NMR (126 MHz, DMSO-d_6) δ 167.34, 162.53, 131.45, 116.05, 60.97, 17.50, 14.67.

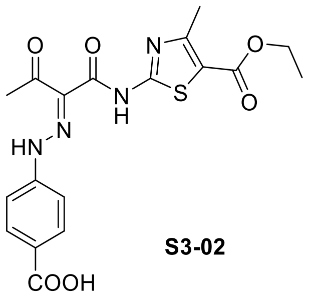

S3-02

(E)-3-(2-(1-((5-(ethoxycarbonyl)-4-methylthiazol-2-yl)amino)-1,3-dioxobutan-2-ylidene)hydrazinyl)benzoic acid: S3-03

S3-03 was synthesized according to the general procedure as yellow solid with 62% yield.

^{1}H NMR (400 MHz, DMSO-*d$_6$*) δ 8.22 (s, 1H), 7.94 (d, *J* = 8.0 Hz, 1H), 7.83 (d, *J* = 8.0 Hz, 1H), 7.43 (t, *J* = 8.0 Hz, 1H), 4.33 (q, *J* = 4.0 Hz, 2H), 2.65 (s, 3H), 2.60 (s, 3H), 1.37 (t, *J* = 4.0 Hz, 3H).

^{13}C NMR (100 MHz, DMSO-*d$_6$*) δ 198.55, 167.22, 162.30, 161.14, 157.91, 142.65, 132.64, 130.36, 127.41, 126.28, 120.94, 117.33, 115.67, 61.18, 26.05, 17.41, 14.61.

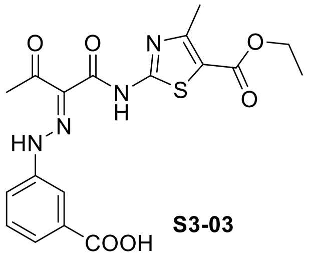

S3-03

(E)-2-(2-(1-((5-(ethoxycarbonyl)-4-methylthiazol-2-yl)amino)-1,3-dioxobutan-2-ylidene)hydrazinyl)terephthalic acid: S3-04

S3-04 was synthesized according to the general procedure as yellow solid with 40% yield.

^{1}H NMR (400 MHz, DMSO-*d$_6$*) δ 12.69 (bs, 1H), 8.36 (s, 1H), 8.06 (d, *J* = 8.0 Hz, 1H), 7.69 (dd, *J* = 8.0 Hz and 4.0 Hz, 1H), 4.24 (q, *J* = 4.0 Hz, 2H), 2.56 (s, 3H), 2.55(s, 3H), 1.30 (t, *J* = 4.0 Hz, 3H).

^{13}C NMR (100 MHz, DMSO-*d$_6$*) δ 199.00, 168.05, 167.42, 162.44, 159.72, 158.45, 156.95, 144.51, 132.21, 128.90, 126.43, 124.79, 115.62, 61.03, 26.21, 17.40, 14.65.

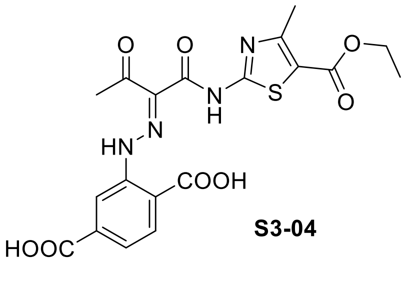

S3-04

(E)-2-(2-(1-((5-(ethoxycarbonyl)-4-methylthiazol-2-yl)amino)-1,3-dioxobutan-2-ylidene)hydrazinyl)-5-hydroxybenzoic acid: S3-05

S3-05 was synthesized according to the general procedure as brown solid with 70% yield.

1H NMR (500 MHz, DMSO-d6) δ 12.89 (s, 1H), 9.69 (s, 1H), 7.68 (d, J = 10.0 Hz, 1H), 7.44 (s, 1H), 6.91 (d, J = 10.0 Hz, 1H), 4.25 (q, J = 5.0 Hz, 2H), 2.55 (s, 3H), 2.51(s, 3H), 1.30 (t, J = 5.0 Hz, 3H).

13C NMR (126 MHz, DMSO-d6) δ 198.87, 168.85, 162.52, 157.00, 155.66, 137.24, 128.12, 123.49, 118.85, 117.57, 116.51, 114.68, 60.95, 26.39, 17.43, 14.68.

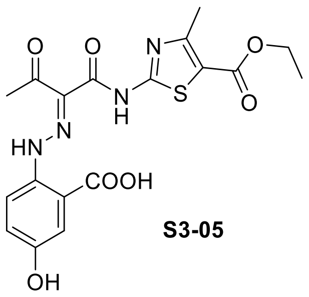

S3-05

Ethyl (E)-2-(2-(2-(2-hydroxyphenyl)hydrazono)-3-oxobutanamido)-4-methylthiazole-5-carboxylate: S3-06

S3-06 was synthesized according to the general procedure as yellow solid with 88% yield.

^{1}H NMR (500 MHz, DMSO-d_6) δ 14.09 (s, 1H), 12.70 (s, 1H), 10.66 (s, 1H), 7.65 (d, *J* = 10.0 Hz, 1H), 7.12 (t, *J* = 10.0 Hz, 1H), 7.02 (d, *J* = 10.0 Hz, 1H), 6.96 (t, *J* = 10.0 Hz, 1H), 4.26 (q, *J* = 5.0 Hz, 2H), 2.57 (s, 3H), 2.54 (s, 3H), 1.31 (t, *J* = 5.0 Hz, 3H).

^{13}C NMR (126 MHz, DMSO-d_6) δ 199.31, 162.27, 161.58, 157.02, 147.20, 129.52, 127.24, 120.76, 116.43, 115.78, 115.48, 61.19, 31.16, 26.21, 17.41, 14.66.

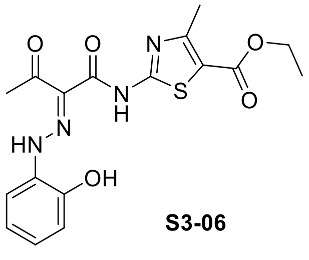

S3-06

Ethyl (E)-2-(2-(2-(2-(hydroxymethyl)phenyl)hydrazono)-3-oxobutanamido)-4-methylthiazole-5-carboxylate: S3-07

S3-07 was synthesized according to the general procedure as yellow solid with 88% yield.

^{1}H NMR (500 MHz, DMSO-d_6) δ 14.34 (s, 1H), 12.64 (s, 1H), 7.79 (d, *J* = 10.0 Hz, 1H), 7.45 (t, *J* = 10.0 Hz, 1H), 7.36 (d, *J* = 10.0 Hz, 1H), 7.22 (t, *J* = 10.0 Hz, 1H), 4.71 (d, *J* = 5.0 Hz, 2H), 4.26 (q, *J* = 5.0 Hz, 2H), 2.57 (s, 3H), 2.55 (s, 3H), 1.30 (t, *J* = 5.0 Hz, 3H).

^{13}C NMR (126 MHz, DMSO-d_6) δ 199.58, 162.31, 160.87, 157.64, 157.04, 140.90, 130.17, 129.37, 129.28, 126.00, 125.49, 116.23, 115.71, 62.14, 61.18, 26.36, 17.42, 14.70.

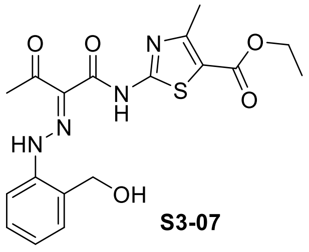

S3-07

(E)-2-(2-(1-((4-methylthiazol-2-yl)amino)-1,3-dioxobutan-2-ylidene)hydrazinyl)benzoic acid: S3-08

S3-08 was synthesized according to the general procedure as yellow solid with 57% yield.

^{1}H NMR (400 MHz, DMSO-d_6) δ 14.18 (s, 1H), 13.20 (bs, 1H), 7.98 (d, *J* = 8.0 Hz, 1H), 7.92 (d, *J* = 8.0 Hz, 1H), 7.68 (t, *J* = 8.0 Hz, 1H), 7.23 (s, 1H), 7.17 (t, *J* = 8.0 Hz, 1H), 2.60 (s, 3H), 2.37 (s, 3H).

^{13}C NMR (100 MHz, DMSO-d_6) δ 198.75, 170.76, 169.12, 150.93, 144.68, 135.01, 131.78, 113.87,

17.71.

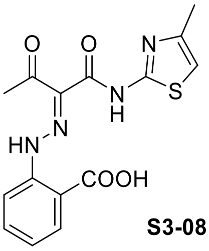

S3-08

(E)-2-(2-(1,3-dioxo-1-(thiazol-2-ylamino)butan-2-ylidene)hydrazinyl)benzoic acid: S3-09

S3-09 was synthesized according to the general procedure as yellow solid with 65% yield.

^{1}H NMR (500 MHz, DMSO-d_6) δ 15.69 (s, 1H), 12.48 (bs, 1H), 8.02 (d, *J* = 10.0 Hz, 1H), 7.92 (d, *J* = 10.0 Hz, 1H), 7.62 (d, *J* = 10.0 Hz, 1H), 7.59 (t, *J* = 10.0 Hz, 1H), 7.55 (t, *J* = 10.0 Hz, 1H), 7.22 (d, *J* = 10.0 Hz, 1H), 2.57 (s, 3H).

^{13}C NMR (126 MHz, DMSO-d_6) δ 199.45, 168.88, 159.60, 156.55, 143.88, 138.61, 133.32, 131.90, 126.97, 124.57, 115.40, 115.03, 26.49.

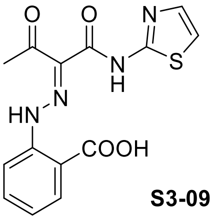

S3-09

(E)-2-(2-(1-((5-(ethoxycarbonyl)-4-methylthiazol-2-yl)amino)-1,3-dioxobutan-2-ylidene)hydrazinyl)-4-methylbenzoic acid: S3-10

S3-10 was synthesized according to the general procedure as yellow solid with 56% yield.

^{1}H NMR (500 MHz, DMSO-d_6) δ 15.18 (s, 1H), 12.57 (bs, 1H), 7.91 (d, *J* = 10.0 Hz, 1H), 7.78 (s, 1H), 7.12 (d, *J* = 5.0 Hz, 1H), 4.27 (q, *J* = 10.0 Hz, 2H), 2.59 (s, 3H), 2.58 (s, 3H), 2.44 (s, 3H), 1.31 (t, *J* = 10.0 Hz, 3H).

^{13}C NMR (126 MHz, DMSO-d_6) δ 156.85, 125.99, 115.94, 61.19, 26.52, 22.06, 17.42, 14.67.

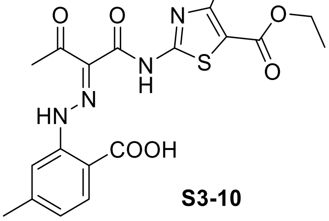

S3-10

(E)-2-(2-(1-((5-(ethoxycarbonyl)-4-methylthiazol-2-yl)amino)-1,3-dioxobutan-2-ylidene)hydrazinyl)-3-methylbenzoic acid: S3-11

S3-11 was synthesized according to the general procedure as yellow solid with 65% yield.

^{1}H NMR (500 MHz, DMSO-d_6) δ 12.77 (bs, 1H), 7.82 (s, 1H), 7.71 (s, 1H), 7.31 (d, *J* = 10.0 Hz, 1H), 4.22 (q, *J* = 5.0 Hz, 2H), 2.51 (s, 3H), 2.32 (s, 3H), 2.17 (s, 3H), 1.26 (t, *J* = 5.0 Hz, 3H).

^{13}C NMR (126 MHz, DMSO-d_6) δ 195.28, 159.53, 151.16, 142.87, 132.19, 114.87, 60.99, 26.39, 17.40, 14.60.

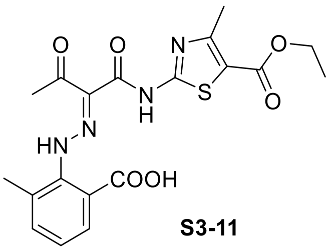

S3-11

Sodium (E)-2-(2-(1-((4-methylthiazol-2-yl)amino)-1,3-dioxobutan-2-ylidene)hydrazinyl)benzoate: S3-12

Compound **S3-08** (1 eq.) and NaOH (1 eq.) were added into *t*-BuOH, stirred at 120℃ for 8h. Removing the solvent to afford desired compounds. Yellow solid with 95% yield. ^{1}H NMR (500 MHz, DMSO-d_6) δ 12.85 (s, 1H), 7.80 (s, 1H), 7.71 (d, *J* = 10.0 Hz, 2H), 7.28 (d, *J* = 10.0 Hz, 2H), 2.56 (s, 3H), 2.51 (s, 3H). ^{13}C NMR (126 MHz, DMSO-d_6) δ 132.19, 114.72, 60.99, 26.43, 21.09, 17.43, 14.69.

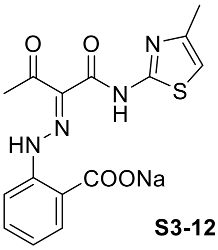

S3-12

(E)-2-(2-(1-((5-(ethoxycarbonyl)-4-methylthiazol-2-yl)amino)-1,3-dioxobutan-2-ylidene)hydrazinyl)-5-methylbenzoic acid: S3-13

S3-13 was synthesized according to the general procedure as yellow solid with 47% yield.

^{1}H NMR (400 MHz, DMSO-d_6) δ 12.88 (s, 1H), 7.80 (s, 1H), 7.71 (d, *J* = 4.0 Hz, 1H), 7.28 (d, *J* = 4.0 Hz, 1H), 4.24 (q, *J* = 8.0 Hz, 2H), 2.56 (s, 3H), 2.53 (s, 3H), 2.32 (s, 3H), 1.30 (t, *J* = 8.0 Hz, 3H).

^{13}C NMR (100 MHz, DMSO-d_6) δ 198.97, 162.52, 156.98, 134.14, 132.37, 132.14, 114.77, 60.97, 26.42, 21.08, 17.42, 14.68.

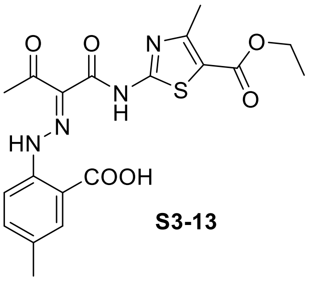

S3-13

(E)-2-(2-(1-((5-(ethoxycarbonyl)-4-methylthiazol-2-yl)amino)-1,3-dioxobutan-2-ylidene)hydrazinyl)-5-fluorobenzoic acid: S3-14

S3-14 was synthesized according to the general procedure as yellow solid with 54% yield.

^{1}H NMR (400 MHz, DMSO-d_6) δ 12.69 (s, 1H), 7.82 (dd, *J* = 4.0 Hz and 8.0 Hz, 1H), 7.68 (d, *J* = 4.0 Hz and 8.0 Hz, 1H), 7.31 (td, *J* = 4.0 Hz and 8.0 Hz, 1H), 4.24 (q, *J* = 8.0 Hz, 2H), 2.56 (s, 3H), 2.53 (s, 3H), 1.30 (t, *J* = 8.0 Hz, 3H).

^{13}C NMR (100 MHz, DMSO-d_6) δ 199.21, 167.43, 162.43, 159.63, 158.38, 156.95, 140.80, 127.97, 125.39, 118.64, 117.55, 116.95, 115.00, 61.02, 26.41, 17.39, 14.65.

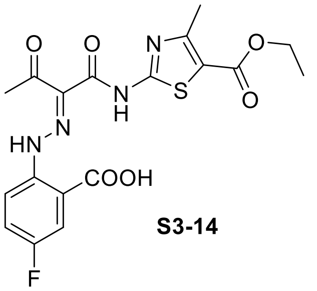

S3-14

(E)-2-(2-(1,3-dioxo-1-(pyridin-2-ylamino)butan-2-ylidene)hydrazinyl)benzoic acid: S3-15

S3-15 was synthesized according to the general procedure as yellow solid with 70% yield.

^{1}H NMR (500 MHz, DMSO-d_6) δ 11.62 (s, 1H), 8.36 (bs, 1H), 8.25 (d, *J* = 5.0 Hz, 1H), 7.97 (d, *J* = 10.0 Hz, 1H), 7.84 (t, *J* = 10.0 Hz, 1H), 7.79 (d, *J* = 10.0 Hz, 1H), 7.42 (t, *J* = 10.0 Hz, 1H), 7.16-7.09 (m, 1H), 2.55 (s, 3H).

^{13}C NMR (126 MHz, DMSO-d_6) δ 148.84, 138.73, 120.22, 114.46, 26.66.

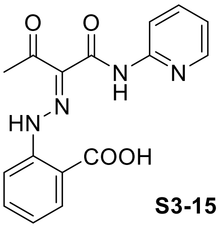

S3-15

(E)-2-(2-(1-(benzo[d]thiazol-2-ylamino)-1,3-dioxobutan-2-ylidene)hydrazinyl)benzoic acid: S3-16

S3-16 was synthesized according to the general procedure as yellow solid with 50% yield.

^{1}H NMR (500 MHz, DMSO-d_6) δ 12.87 (s, 1H), 8.00 (d, *J* = 10.0 Hz, 1H), 7.82 (d, *J* = 5.0 Hz, 1H), 7.78 (d, *J* = 10.0 Hz, 1H), 7.47 (q, *J* = 5.0 Hz, 2H), 7.33 (t, *J* = 5.0 Hz, 1H), 7.17 (t, *J* = 10.0 Hz, 1H), 2.58 (s, 3H).

^{13}C NMR (126 MHz, DMSO-d_6) δ 199.17, 168.73, 149.12, 145.46, 132.28, 131.93, 131.37, 126.62, 124.00, 122.15, 120.99, 114.61, 26.52.

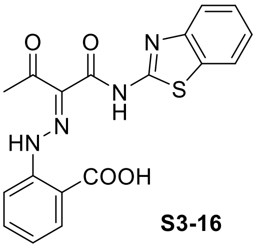

S3-16

(E)-2-(2-(1-(3,4-dihydroisoquinolin-2(1H)-yl)-1,3-dioxobutan-2-ylidene)hydrazinyl)benzoic acid: S3-17

S3-17 was synthesized according to the general procedure as yellow solid with 56% yield.

^{1}H NMR (500 MHz, DMSO-d_6) δ 15.29 (s, 1H), 15.18 (s, 1H), 8.36 (bs, 1H), 7.87 (t, *J* = 10.0 Hz, 1H), 7.60 (d, *J* = 5.0 Hz, 1H), 7.34-7.09 (m, 5H), 7.84 (t, *J* = 10.0 Hz, 1H), 6.90 (t, *J* = 10.0 Hz, 1H), 4.41 (s, 1H), 3.84 (s, 1H), 3.44 (t, *J* = 5.0 Hz, 1H), 3.44 (s, 2H), 2.76 (t, *J* = 5.0 Hz, 1H), 2.43 (s, 3H).

^{13}C NMR (126 MHz, DMSO-d_6) δ 194.57, 194.38, 170.11, 164.04, 145.10, 145.02, 138.62, 138.57, 134.69, 133.07, 131.86, 130.95, 129.03, 128.92, 127.02, 126.93, 126.78, 126.61, 126.50, 123.42, 120.95, 112.70, 112.64, 46.48, 43.47, 43.21, 29.61, 28.30, 24.79, 24.76.

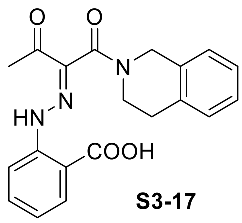

S3-17

(E)-2-(2-(1-morpholino-1,3-dioxobutan-2-ylidene)hydrazinyl)benzoic acid: S3-18

S3-18 was synthesized according to the general procedure as white solid with 81% yield.^{1}H NMR (500 MHz, DMSO-d_6) δ 12.87 (s, 1H), 8.00 (d, *J* = 0.0 Hz, 1H), 7.82 (d, *J* = 5.0 Hz, 1H), 7.78 (d, *J* = 10.0 Hz, 1H), 7.47 (q, *J* = 5.0 Hz, 2H), 7.33 (t, *J* = 5.0 Hz, 1H), 7.17 (t, *J* = 10.0 Hz, 1H), 2.58 (s, 3H), ^{13}C NMR (126 MHz, DMSO-d_6) δ 194.40, 163.22, 144.96, 138.21, 131.92, 131.09, 121.03, 112.77, 66.71, 66.03, 46.24, 41.60, 24.69

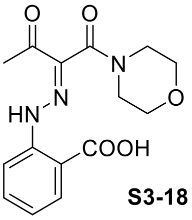

S3-18

(E)-2-(2-(1-((5-(ethoxycarbonyl)-4-methylthiazol-2-yl)amino)-1,3-dioxobutan-2-ylidene)hydrazinyl)isophthalic acid: S3-19

S3-19 was synthesized according to the general procedure as orange solid with 66% yield.

^{1}H NMR (500 MHz, DMSO-d_6) δ 7.79-7.78 (bs, 2H), 7.21 (t, *J* = 5.0 Hz, 1H), 4.26 (q, *J* = 5.0 Hz, 2H), 2.57 (s, 3H), 2.41 (s, 3H), 1.30 (t, *J* = 5.0 Hz, 3H).

^{13}C NMR (126 MHz, DMSO-d_6) δ 199.50, 169.29, 162.52, 159.74, 158.73, 156.94, 143.49, 132.33, 126.86, 126.16, 124.07, 114.89, 60.97, 26.42, 17.42, 14.68.

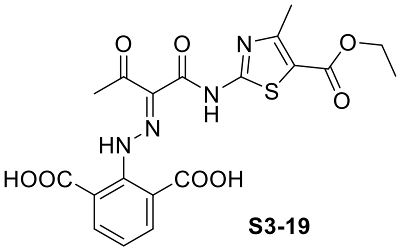

S3-19

(E)-2-(2-(1-((4-methyl-5-(morpholine-4-carbonyl)thiazol-2-yl)amino)-1,3-dioxobutan-2-ylidene)hydrazinyl)benzoic acid: S3-20

S3-20 was synthesized according to the general procedure as yellow solid with 32% yield.

^{1}H NMR (500 MHz, DMSO-d_6) δ 15.06 (bs, 1H), 7.90 (d, *J* = 10.0 Hz, 1H), 7.59 (d, *J* = 10.0 Hz, 1H), 7.34 (t, *J* = 10.0 Hz, 1H), 6.91 (t, *J* = 10.0 Hz, 1H), 3.73-3.53 (m, 8H), 3.19 (s, 3H), 2.40 (s, 3H).

^{13}C NMR (126 MHz, DMSO-d_6) δ 194.39, 170.08, 163.22, 144.99, 138.17, 131.90, 131.08, 123.08, 121.02, 112.73, 66.70, 65.99, 46.22, 41.57, 24.70.

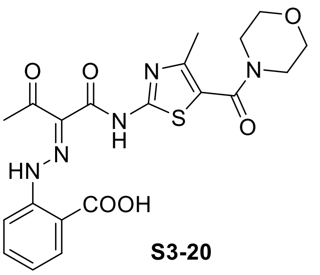

S3-20

(E)-2-(2-(1-((5-(ethoxycarbonyl)-4-methylthiazol-2-yl)amino)-1,3-dioxobutan-2-ylidene)hydrazinyl)-5-methoxybenzoic acid: S3-21

S3-21 was synthesized according to the general procedure as red solid with 61% yield.

^{1}H NMR (500 MHz, DMSO-d_6) δ 12.86 (s, 1H), 7.76 (d, J = 10.0 Hz, 1H), 7.54 (s, 1H), 7.06 (d, J = 10.0 Hz, 1H), 4.25 (q, J = 5.0 Hz, 2H), 3.80 (s, 3H), 2.57 (s, 3H), 2.53 (s, 3H), 1.31 (t, J = 5.0 Hz, 3H).

^{13}C NMR (126 MHz, DMSO-d_6) δ 198.89, 162.53, 157.05, 157.00, 118.25, 116.40, 115.11, 60.97, 55.73, 26.40, 17.42, 14.68.

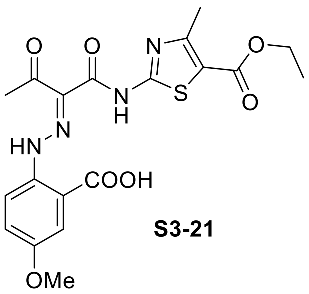

S3-21

(Z)-2-(2-(1-((5-(ethoxycarbonyl)-4-methylthiazol-2-yl)amino)-3-isopropoxy-1,3-dioxopropan-2-ylidene)hydrazinyl)benzoic acid: S3-22

S3-22 was synthesized according to the general procedure as yellow solid with 62% yield.

^{1}H NMR (500 MHz, DMSO-d_6) δ 12.64 (s, 1H), 8.02 (d, J = 10.0 Hz, 1H), 7.82 (d, J = 10.0 Hz, 1H), 7.48 (t, J = 10.0 Hz, 1H), 7.17 (t, J = 10.0 Hz, 1H), 3.62 (d, J = 5.0 Hz, 3H), 3.54 (d, J = 5.0 Hz, 3H), 2.56 (s, 3H), 2.29 (t, J = 5.0 Hz, 3H).

^{13}C NMR (126 MHz, DMSO-d_6) δ 199.09, 169.45, 162.85, 159.53, 156.27, 147.84, 144.72, 132.01, 131.62, 124.48, 118.58, 114.76, 66.67, 26.43, 16.77.

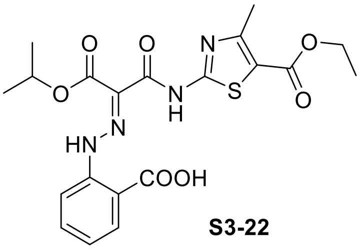

S3-22

(E)-2-(2-(1-((5-cyanopyridin-2-yl)amino)-1,3-dioxobutan-2-ylidene)hydrazinyl)benzoic acid: S3-15-1

S3-15-1 was synthesized according to the general procedure as yellow solid with 55% yield.

^{1}H NMR (500 MHz, DMSO-d_6) δ 12.02 (s, 1H), 8.77 (s, 1H), 8.34 (d, J = 10.0 Hz, 1H), 8.24 (s, 1H), 7.98 (d, J = 10.0 Hz, 1H), 7.78 (d, J = 10.0 Hz, 1H), 7.44 (t, J = 10.0 Hz, 1H), 7.14 (t, J = 10.0 Hz, 1H), 2.54 (s, 3H).

^{13}C NMR (126 MHz, DMSO-d_6) δ 199.37, 169.17, 160.97, 154.17, 152.74, 143.83, 142.50, 132.03, 131.24, 126.41, 124.42, 117.68, 114.64, 113.78, 104.09, 26.65.

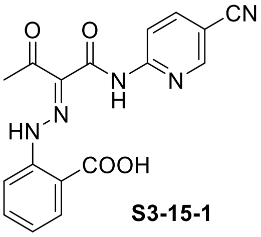

S3-15-1

(E)-2-(2-(1-((6-(methoxycarbonyl)pyridin-2-yl)amino)-1,3-dioxobutan-2-ylidene)hydrazinyl)benzoic acid: S3-15-2

S3-15-2 was synthesized according to the general procedure as yellow solid with 34% yield.

^{1}H NMR (500 MHz, DMSO-d_6) δ 11.72 (s, 1H), 8.45 (d, J = 10.0 Hz, 1H), 8.34 (d, J = 10.0 Hz, 1H), 8.05 (t, J = 10.0 Hz, 1H), 7.96 (d, J = 10.0 Hz, 1H), 7.82-7.79 (m, 2H), 7.43 (t, J = 10.0 Hz, 1H), 7.11 (t, J = 10.0 Hz, 1H), 3.90 (s, 3H), 2.55 (s, 3H).

^{13}C NMR (126 MHz, DMSO-d_6) δ 212.17, 193.55, 165.24, 158.59, 146.52, 140.16, 131.98, 118.13, 106.07, 94.02, 83.93, 52.94, 26.61.

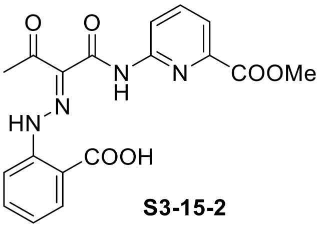

S3-15-2

(E)-2-(2-(1,3-dioxo-1-(pyridin-2-ylamino)butan-2-ylidene)hydrazinyl)-5-hydroxybenzoic acid: S3-15-3

S3-15-3 was synthesized according to the general procedure as yellow solid with 71% yield.

^{1}H NMR (500 MHz, DMSO-d_6) δ 11.79 (s, 1H), 9.77 (s, 1H), 8.32 (d, J = 10.0 Hz, 1H), 8.23 (d, J = 10.0 Hz, 1H), 7.76 (t, J = 10.0 Hz, 1H), 7.66 (d, J = 10.0 Hz, 1H), 7.10 (t, J = 10.0 Hz, 1H), 6.91 (d, J = 10.0 Hz, 1H), 2.51 (s, 3H).

^{13}C NMR (126 MHz, DMSO-d_6) δ 198.96, 169.44, 161.18, 155.17, 151.66, 148.76, 138.63, 135.70, 128.30, 124.73, 120.07, 118.50, 117.90, 116.32, 114.38, 26.64.

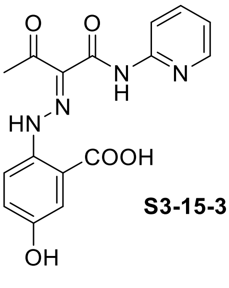

S3-15-3

(Z)-2-(2-(1-isopropoxy-1,3-dioxo-3-(pyridin-2-ylamino)propan-2-ylidene)hydrazinyl)benzoic acid: S3-15-4

S3-15-4 was synthesized according to the general procedure as yellow solid with 68% yield.

^{1}H NMR (500 MHz, DMSO-d_6) δ 11.27 (s, 1H), 8.35 (s, 1H), 8.24-8.12 (m, 1H), 7.97-7.92 (m, 1H), 7.84 (t, J = 10.0 Hz, 1H), 7.72 (t, J = 10.0 Hz, 1H), 7.42-7.35 (m, 1H), 7.16 (t, J = 10.0 Hz, 1H), 7.07-6.95 (m, 1H), 5.19-5.11 (m, 1H), 1.34 (s, 6H).

^{13}C NMR (126 MHz, DMSO-d_6) δ 169.43, 151.59, 148.80, 148.58, 138.75, 132.06, 131.89, 130.84, 123.30, 120.31, 114.22, 113.79, 69.43, 68.88, 22.20, 21.96.

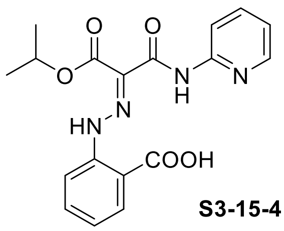

S3-15-4
